# Investigating the psychedelic hypothesis of *kykeon*, the sacred elixir of the Eleusinian Mysteries

**DOI:** 10.1038/s41598-026-39568-3

**Published:** 2026-02-13

**Authors:** Romanos K. Antonopoulos, Evangelos Dadiotis, Kostas Ioannidis, Antigoni Cheilari, Vangelis Mitsis, Ana M. Garcia-Campaña, Laura Gámiz-Gracia, Maykel Hernández-Mesa, Alfonso Narváez, Mark A. Hoffman, Carl A. P. Ruck, Zacharoula Gonou-Zagou, Nektarios Aligiannis, Prokopios Magiatis

**Affiliations:** 1 Laboratory of Pharmacognosy and Chemistry of Natural Products, Department of Pharmacy, National and Kapodistrian University of Athens (NKUA), Panepistimioupoli Zografou, Athens, 15771 Greece; 2 Hellenic Center for Entheogenic Research, NPC, Porto Rafti, Attica, 19003 Greece; 3 Laboratory of Sylviculture, Forest Genetics and Biotechnology, Institute of Mediterranean and Forest Ecosystems, Hellenic Agricultural Organization “Demeter”, Ilissia, Athens, 11528 Greece; 4 Department of Analytical Chemistry, Faculty of Sciences, University of Granada, Granada, 18071 Spain; 5 The Wasson Ruck Entheogenic Research Institute and Archives, Taos, NM 87571 USA; 6 Department of Classical Studies, Boston University, Boston, MA 02215 USA; 7 Section of Ecology and Systematics, Department of Biology, National and Kapodistrian University of Athens (NKUA), Panepistimioupoli Zografou, Athens, 15784 Greece

**Keywords:** Psychedelic, *kykeon*, Eleusinian Mysteries, *Claviceps purpurea*, Ergot alkaloids, Lysergic acid amide (LSA), Biochemistry, Chemistry

## Abstract

This study revisits the hypothesis that *Claviceps purpurea* (Fr.) Tul., a fungus infecting cereals and producing ergot alkaloids (EAs), was the psychedelic agent in *kykeon*, the sacred elixir of the Eleusinian Mysteries. Although archaeological evidence supports this link, experimental data confirming the transformation of toxic ergopeptides into psychoactive lysergic acid amide derivatives in putative ancient preparations remain limited. The potential hydrolysis products of ergot were investigated using a technique that could have been readily applied by the priestesses of Eleusis, notably reacting in lye. Pulverized sclerotia were refluxed in solutions of lye and in distilled water. Samples obtained after liquid-liquid extractions were analyzed with ^1^H-NMR and UHPLC/Q-TOF-HRMS. The ^1^H NMR spectra demonstrated conversion of toxic ergopeptides, present in control samples but absent at all reaction times in 5% w/v ergot treated with pH 12.5 lye, where characteristic peaks for lysergic acid amide (LSA; ergine) and isolysergic acid amide (iso-LSA; isoergine; erginine) appeared. UHPLC/Q-TOF-HRMS quantitation confirmed this conversion, yielding 0.54 mg LSA and 0.48 mg iso-LSA per gram of ergot at 120 min. The chemical transformation of toxic EAs to psychoactive compounds, utilizing ancient technology, was demonstrated and supports the “psychedelic Eleusis” hypothesis.

**Supplementary Information:**

The online version contains supplementary material available at 10.1038/s41598-026-39568-3.

## Introduction

Psychedelics, a diverse class of psychoactive substances, intertwined with human spirituality since prehistory, are re-emerging as tools for treating neuropsychiatric disorders and exploring consciousness^1^. While contemporary studies rapidly expand their clinical perspectives^1^, ritual use of psychedelic preparations provides valuable insights into the cultural frameworks shaping their effects^2^. Situating modern psychedelic research within this broader historical continuum can enrich current therapeutic approaches by acknowledging the interplay between substance and setting while drawing on ancestral ethnopharmacological knowledge^2^. In this context, the hypothesis of the psychedelic ceremonial use of *kykeon*, the sacred elixir marking the climax of the Eleusinian Mysteries^3^, is revisited.

These rites, known in antiquity as *Μυστήρια τὰ ἐν Ἐλευσῖνι*, were annual autumnal agrarian religious festivals culminating in the initiation hall (*Τελεστήριον*) of Eleusis (*Ἐλευσίς*, modern Elefsina), 20 km west of Athens via the Holy Way (*Ἱερὰ Ὁδός*)^3^. Conducted for nearly two millennia, they attracted people throughout the Mediterranean, constituting the most important esoteric tradition of Classical antiquity^3,4^. Rich in symbolism, tied to the life-death-rebirth cycle, they exerted lasting influence on Western tradition^3^. Through initiation into collective experience, they transcended divisions of gender, class, and ethnicity, conveying a universal message^4^. Pausanias praised them as one of Greece’s greatest wonders, alongside the Olympic Games, both safeguarded by the providence of the Gods^3^.

The mythological basis of the cult lies in a narrative rooted in the 4th millennium, first recorded around the 7th century BCΕ^3^, in an anonymous epic known as *The Homeric Hymn to Demeter*, part of the Panhellenic hymn tradition^5^. At its core unfolds the story of *Persephone*’s (or *Κόρη*, meaning *“*daughter/maiden”) abduction by *Pluto* (or *Hades*, God of the underworld) and her mother *Demeter*’s (Goddess of nature, harvest, agriculture, and fertility) search for her in Eleusis^5^. These deities were later assimilated into the Roman pantheon through religious syncretism, becoming *Proserpina*, *Dis Pater*, and *Ceres*, a tradition that endured until its suppression by rising Christianity in the 4th century CE^3^.

Central to the experience was the potation of kykeon (*κῠκεών* from the verb *κῠκάω* “to mix”), a ceremonial drink whose composition and use remain a topic of debate^6^. Many scholars view its drinking as a sacramental act of communion^3,4^, others argue that it served as a vehicle for an entheogen^7,8^, suggesting cognateness with the *soma* ritual^9,10^, while some dispute the “psychedelic Eleusis” hypothesis^11–13^. “Entheogen” (from *ἔνθεος*, “God within,” and *γενέσθαι*, “to give birth to”) was coined to describe psychoactive substances that induce altered states of consciousness in ceremonial settings^14^, avoiding the terms “psychedelics” (from *ψυχή* meaning “soul” but also “butterfly”, its emergence from the chrysalis symbolizing the soul’s release at death^15^ and *δῆλος* meaning “to manifest”^16^) or “hallucinogens” which carry cultural biases^14^.

The initiation of consuming the kykeon is described in lines 206–210 of the Hymn^5^. When Metaneira, the queen of Eleusis offered Goddess Demeter “*honey-sweet wine”* to soothe her sorrow, the Goddess refused, replying that *“it is not lawful for her to drink red wine”* but she ordered them *“mix barley and water with soft mint and give her to drink”* and Metaneira *“prepared the kykeon and offered it to the goddess*,* as she bade*”^5^. In practice, within the darkened temple, participants had to recite the formula: “*I have fasted*,* I have drunk the kykeon*”^17^. What happened thereafter is, as the name suggests (Mysteries deriving from *µύω*, “to keep eyes/mouth shut”) an enigma, the penalty for revealing what occurred, in public or private, being death^3^. However, considerable information survives from the myth^5^, the Lesser Eleusinian Mysteries (*Ἐλευσίνια τὰ Μικρά*, public pre-festivals)^3^, organizational records^3^, and polemical early Christian accounts^17^. We can be reasonably certain of their psychedelic core through numerous accounts of the experience, namely ego dissolution and the “resurrection” of the soul into a higher state of being, where psychedelic and religious dimensions likely converged. Pindar and Cicero both link initiation with knowledge of death and afterlife and, by extension, with happiness^3^.

Building on the enduring mystery surrounding kykeon’s composition, scholarship has long sought to identify its psychoactive components^6^. There are many variants of kykeon described in ancient Greek texts (see Supplementary Note 2). With focus on the Eleusinian one, its components are given as water, mint, and barley^5^. While fermented barley and its alcohol content may contribute to the drink’s flavor profile, it would not suffice to induce an entheogenic reaction, with alcohol also being denied by the Goddess. Another psychoactive candidate is mint, referred to in text as *γλήχωνι*, identified as pennyroyal (*Mentha pulegium*), a fragrant herb with known calming properties^4^. Although toxic in high doses, pennyroyal is presumed non-psychedelic^4^. In addition to barley and mint, the opium poppy (*Papaver somniferum*) was closely linked to the Eleusinian cult, depicted on reliefs^12^. However, opium was well known in ancient Greek medicine and rituals^18^, thus its public symbolism and narcotic-sedative profile argue against it being the hidden entheogen^12^. Other candidates, including the psychedelic *Amanita muscaria* and psilocybin mushrooms remain speculative^13^, due to their seasonal nature, irregular availability, and non-cultivability, making their use unlikely in a structured ritual involving thousands of initiates.

**Fig. 1:**
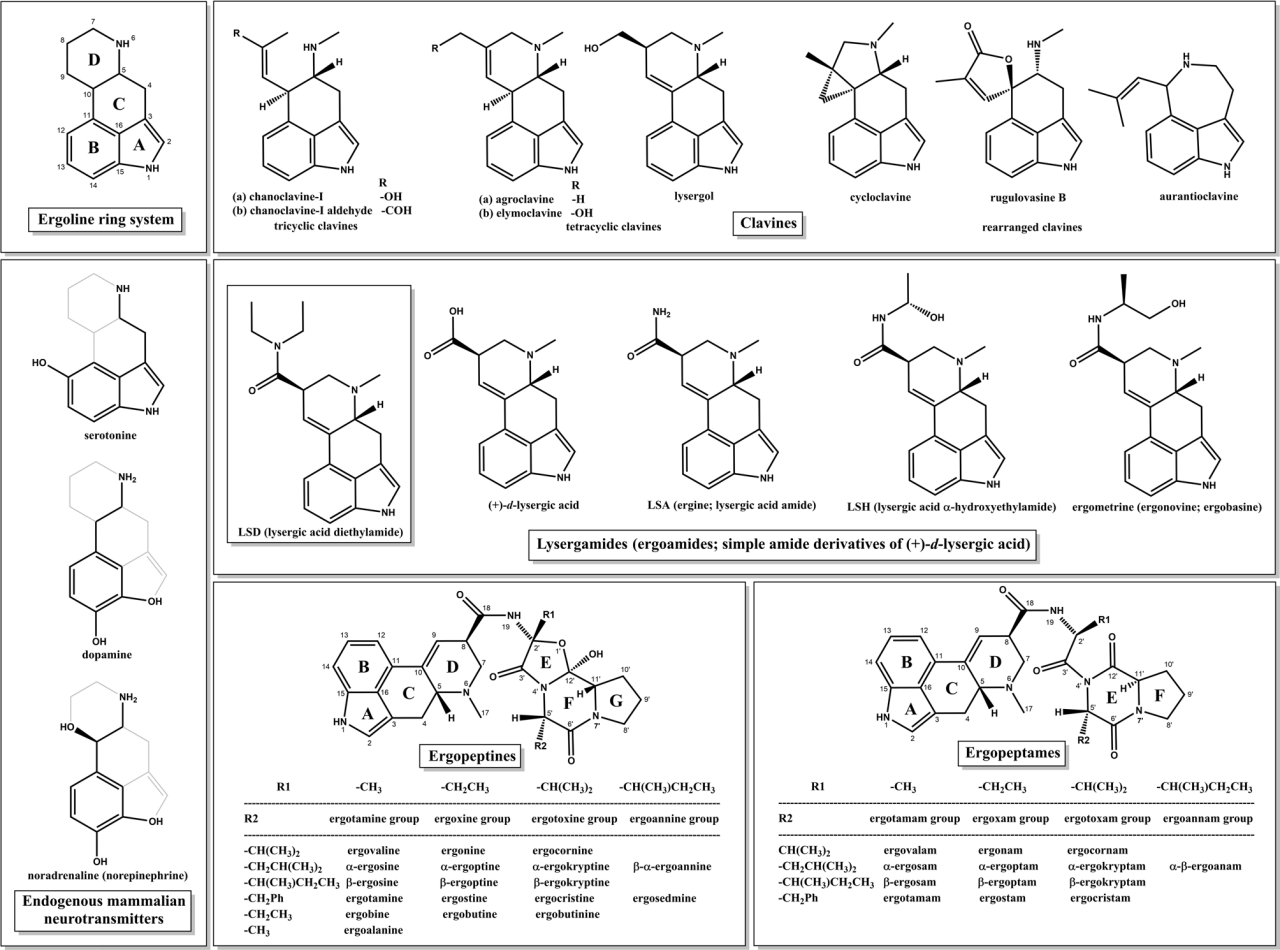
The four biosynthetic classes of EAs, LSD, and related endogenous neurotransmitters. Top left: the core structure of the tetracyclic ergoline ring system. Lower left: superimposition of the endogenous mammalian neurotransmitters serotonin, dopamine, and noradrenaline onto the general ergoline alkaloid structure. Upper right: representative structures of the clavine-type EAs. Middle: lysergic acid diethylamide (LSD), one of the most potent semisynthetic psychedelic drugs, and simple lysergic acid derivatives (lysergamides; ergoamides). Bottom center: ergopeptines. Bottom right: ergopeptames. For detailed descriptions on EAs structures and stereochemistry, see Supplementary Note 1. *The C-8 epimers are not depicted here for the sake of brevity*.

The 1970s marked a turning point with *The Road to Eleusis: Unveiling the Secret of the Mysteries*^8^ by R. Gordon Wasson, the mycologist who introduced “magic” mushrooms to the West^19^, Albert Hofmann, the chemist who synthesized LSD^20^ (Fig. 1) and Carl A. P. Ruck, the classicist who coined the term “entheogen”^14^. They advanced the hypothesis that ergot alkaloids (EAs), produced by *Claviceps purpurea*, a plant-parasitic ascomycete filamentous fungus, were the secret entheogenic agents in kykeon^8^. Recent archaeological evidence from a ritual site in Mas Castellar de Pontós, a city near *Ἐµπόριον* (meaning “trade”), an ancient Greek colony and major cereal trading port^21^, lends support to this theory. Inside a sanctuary dedicated to the two Eleusinian Goddesses^22^, archaeologists discovered fragments of ergot both inside a ceremonial vase and in the dental calculus of a 25-year-old man, providing evidence of its consumption^23,24^. Ergot-infected barley is considered the most probable candidate among the hypothesized psychoactive ingredients in kykeon, as it aligns with the Hymn’ ingredients, plausible pharmacology and archeological findings.

Ergot refers to the compactly interwoven hyphae of the fungus, from the French *argot*, meaning “claw” or “spur”, which reflects its morphology, biologically designated as a *sclerotium*, the fungus’s dormant winter stage, from the Greek *σκληρός*, meaning “hard”^25^. The herbal drug is called *Secale cornutum* and the comminuted sclerotia *Pulvis parturiens* referring to its use in obstetrics until the early 20th century^25^. However, one major concern regarding this hypothesis is ergot’s toxicity. Its effects are manifested as *ergotism*, a disease historically known as *Saint Anthony’s Fire* or *ignis sacer*, with epidemics of tragic dimensions during the Middle Ages, including the 1418 Paris outbreak that claimed 50,000 lives^26^. Symptoms can include hallucinations, severe burning sensations in the limbs, seizures, spasms, convulsions, vomiting, dry gangrene and consequent limb loss due to the intense vasoconstriction, caused by the unintentional consumption of toxic ergopeptides (Fig. 1) through contaminated grains and the foodstuffs made thereof^25^. This raises a critical and still unanswered question: If ergot was indeed an active component of the kykeon, by what means did the ancient Eleusinian priestesses manage to harness its psychoactive potential while avoiding its devastating toxic effects? The answer to this lies in forgotten methods of preparation, precise dosing and the presence of synergistic or detoxifying agents; possibilities modern science has yet to explore.

One suggestion, later proposed^27^ and revisited in the appendix of the 30th anniversary edition of *The Road to Eleusis*^28^, is that the ancient Greeks may have used a simple alkaline preparation made with water and ashes to hydrolyze toxic ergopeptides, into simpler psychedelic lysergamides, such as LSA (Fig. 1) and its C-8 epimer, iso-LSA. Ancient Greeks knew alkali-based medicines^29^, lending plausibility to Eleusinian priestesses empirically developing such a method. The hydrolysis of isolated pure ergopeptides to yield LSA using alcoholic KOH and of LSA to lysergic acid using aqueous NaOH, is well documented^30–34^. However, the conversion of ergopeptides’ mixture of crude ergot sclerotia using a simple lye process, as proposed in the preceding hypothesis, has not been previously examined.

This study examines kykeon in the context of naturalistic psychedelic ceremonial use, identifying ergot as the most likely entheogenic ingredient among the proposed candidates. To this end, acknowledging its toxicity and the lack of experimental data, the potential hydrolysis products of crude, pulverized ergot sclerotia were investigated, using a technique that could have been readily employed by Eleusinian priestesses. The method involved heating in lye, made with water and ashes, as a possible means of detoxification of ergot in kykeon into an entheogenic preparation. To explore and optimize the parameters of this reaction, a full factorial design was applied, allowing the identification of the most favorable conditions for complete hydrolysis through this ancient technique, using analytical tools such as NMR and UHPLC/Q-TOF-HRMS.

## Results

### Spectroscopic and spectrometric data of isolated LSA and iso-LSA

**Fig. 2:**
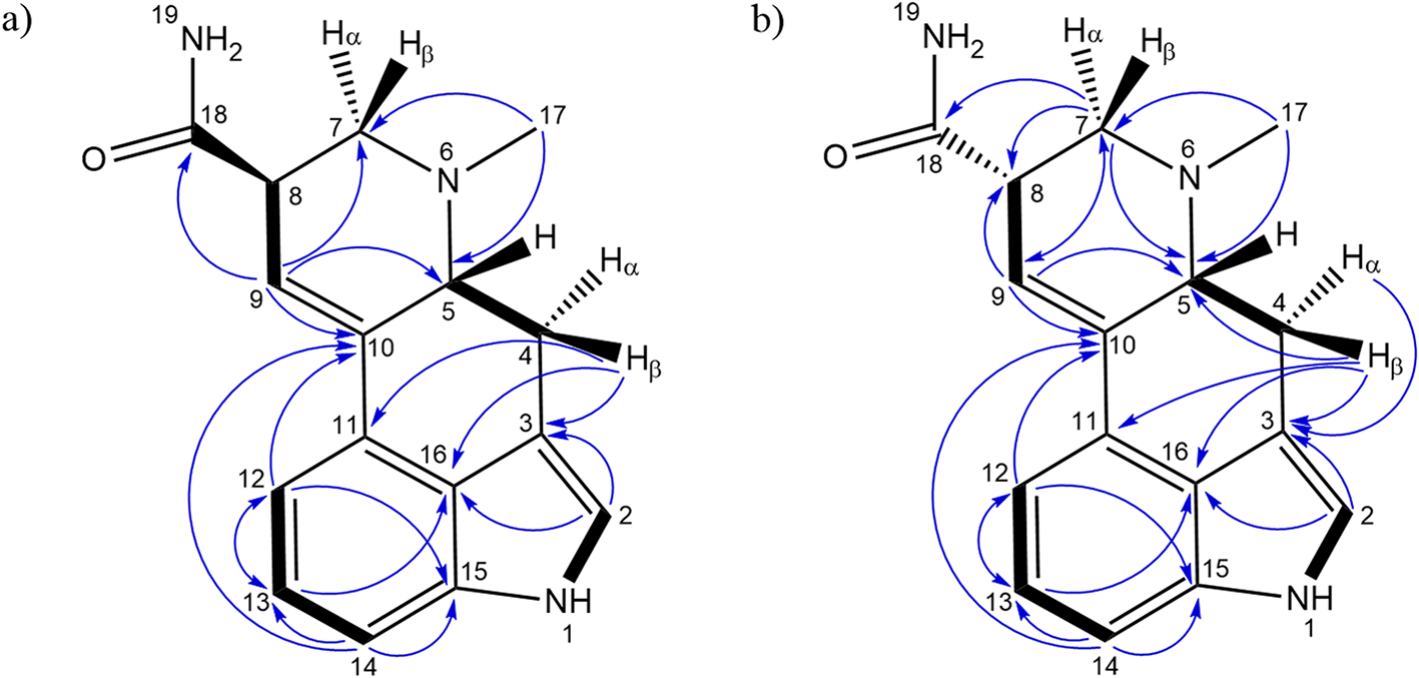
Key^1^H–^1^H COSY (bold lines) and ^1^H–^13^C HMBC (blue arrows) correlations observed for LSA (**a**) and iso-LSA (**b**).

**Fig. 3:**
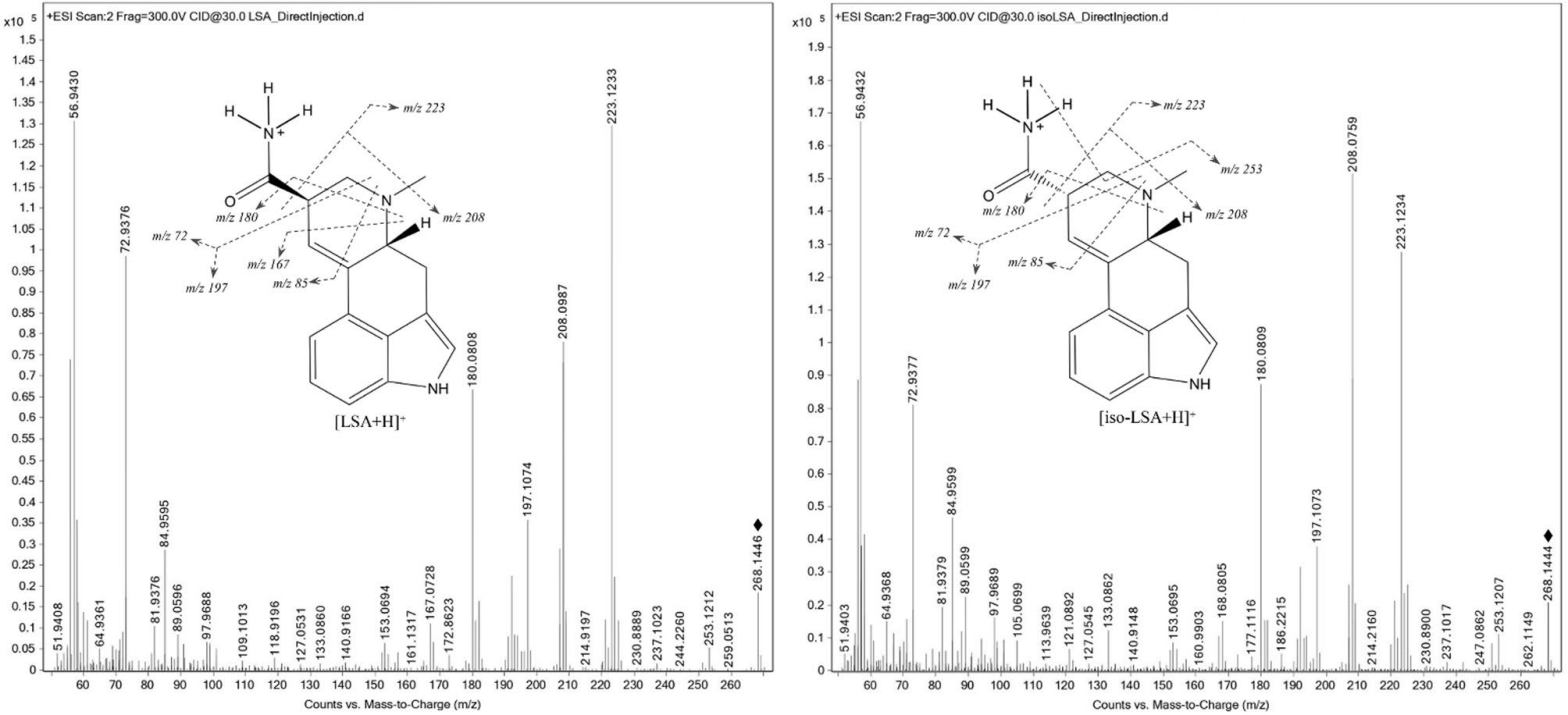
ESI(+)-HRMS fragmentation spectra of LSA and iso-LSA ([M + H]^+^, exact *m/z* 268.1444), showing the proposed major fragment ions. Spectra were obtained by infusion of 0.04 µg mL^-1^ solutions of each analyte in acetonitrile at 0.4 mL min^-1^ into the Agilent 6550 iFunnel Q-TOF Dual AJS ESI mass spectrometer. ♦=*m/z* observed for the [M + H]^+^.

Although LSA and iso-LSA have been known since the 1930s, literature still lacks comprehensive NMR data. Thus, they were isolated and recrystallized from *Argyreia nervosa* seeds. Their ^1^H NMR data in CDCl_3_ and DMSO-d_6_, together with ^13^C NMR data in DMSO-d_6_ are summarized in Table 1. The ^1^H–^1^H COSY and ^1^H–^13^C HMBC correlations are presented in Fig. 2. The 1D and 2D NMR spectra are presented in the Supplementary Fig. S1 to Fig. S22. Their ESI(+)-HRMS fragmentation spectra are shown in Fig. 3 and were consistent with the fragmentation patterns reported in the literature for EAs^35^.

**Table 1:** ^1^H and ^1^^3^C NMR data (400 MHz) for LSA and iso-LSA. Chemical shifts (*δ*, ppm) are referenced to residual solvents peaks. Abbreviations: br, broad (indicative of small unresolved couplings); s, singlet; d, doublet; t, triplet; m, multiplet plus combinations, e.g. dd; doublet of doublets; ov, overlapped; *J* values (Hz) in parenthesis; numbering according to Fig. 1. ^a^in CDCl_3_; ^b^in DMSO-d_6_.

Position	LSA^a^ δ_H_	iso-LSA^a^ δ_H_	LSA^b^ δ_H_	iso-LSA^b^ δ_H_	LSA^b^ δ_C_	iso-LSA^b^ δ_C_
1	7.91, s	8.18, s	10.67, s	10.70, s		
2	6.93, t (1.9)	6.92, t (1.9)	7.02, t (1.8)	7.04, t (1.8)	119.2	119.2
3					108.9	109.0
4α	2.77, m, ov	2.66, m, ov	2.46, m, ov	2.53, m, ov	26.7	26.8
4β	3.40, m, ov	3.57, dd (14.6, 5.6)	3.45, dd (14.8, 5.5)	3.45, dd (14.6, 5.6)		
5	3.56, m	3.17, m, ov	2.97, m	3.03, m	62.6	62.3
6						
7α	3.10, dd (11.6, 4.6)	3.13, m, ov	3.04, dd (11, 5.1)	3.10, dd, (11.6, 2)	55.4	53.9
7β	2.79, m, ov	2.71, dd, ov, (12, 3.9)	2.47, m, ov	2.57, dd, ov, (11.7, 4.2)		
8	3.38, m, ov	3.11, brs, ov	3.38, m	2.98, m	42.5	42.5
9	6.45, dd (4.3, 2)	6.60, brd (6.9)	6.39, brs	6.49, dd (5.6, 1.4)	120.0	119.3
10					127.4	127.7
11					135.1	135.9
12	7.16, m, ov	7.16, m, ov	7.06, m, ov	7.07, m, ov	111.1	111.1
13	7.22, m, ov	7.22, m, ov	7.17, m	7.19, m	109.7	109.8
14	7.17, m, ov	7.17, m, ov	7.06, m, ov	7.07, m, ov	122.3	122.3
15					133.8	133.8
16					125.8	126.0
NCH_3_-17	2.62, s	2.58, s	2.45, s	2.47, s	43.4	43.2
18					173.7	174.6
NH_2_-19	5.28, brs 6.82, brs	5.47, brs 8.03, brs	6.99, brs 7.46, brs	6.97, brs, 7.38, brs		

## Micromorphological and molecular identification of ergot

The field-collected strain’s conidial length and width measurements were consistent with the published ranges for *Claviceps purpurea sensu lato*^36,37^. Molecular analysis of the specimens’ rDNA-ITS region identified it as belonging to *Claviceps purpurea* (Fr.) Tul. The sequence was deposited in GenBank under the accession number PX700742. Indicatively, images of the conidia’s micromorphological characters, images of the sclerotia, images of the inoculated sclerotia and the sequence of the ITS rDNA region of the specimen ATHUM 10382 are presented in the Supplementary Fig. S23 and Table S1.

## Effect of atmospheric neutralization and ergot sclerotia on lye solution pH

The primary lye solution had an initial pH of 12.5. Gradual neutralization by atmospheric CO_2_ reduced its pH to 11.5 and 10.5 after 2 and 5 days, respectively. Adding ergot sclerotia powder further lowered the pH by approximately 2 units. Consequently, experiments were conducted by refluxing ergot sclerotia in lye solutions with final pH ranges of 7.2–8.6, 9.5–10.0, and 10.0–10.8, obtained by the addition of the different concentrations of ergot sclerotia powder to the initial lye solution of pH 10.5, 11.5 and 12.5 respectively. In controls with deionized water, pH dropped to 6.4, 6.2, and 5.8 after adding 5%, 10%, and 20% w/v ergot sclerotia powder, respectively.

## Qualitative analysis of EAs hydrolysis in putative kykeon preparations

**Fig. 4:**
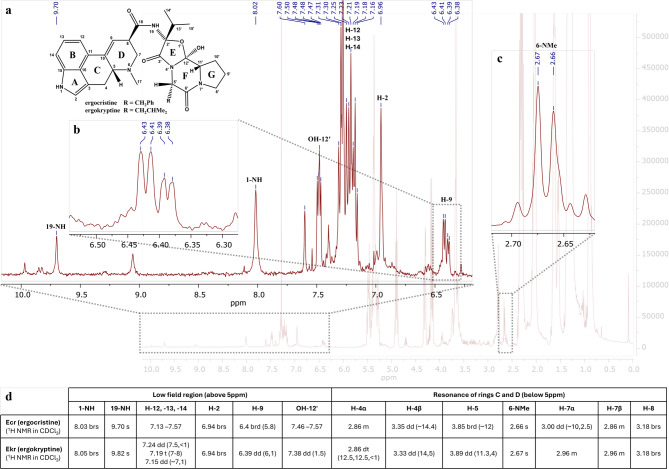
^1^H NMR spectrum in CDCl_3_ of the 5% w/v ergot control sample, refluxed in distilled water, 15 min. Insets (a–c) provide detailed views of characteristic signals for ergopeptides, including amide (19-NH), indole (1-NH), N-methyl (6-NMe), aromatic (H-12,-13,-14), and vinylic (H-9) protons. Inset (d) summarizes chemical shift assignments for the main identified alkaloids, ergocristine (Ecr) and ergokryptine (Ekr), according to literature standards^38^.

Primary monitoring by TLC, following visualization, revealed four main bands of indole alkaloids in the control samples, with R_f_ values of 0.07 (intense purple), 0.33 (faint purple), 0.67 (purple), and 0.83 (faint purple). In contrast, the test samples of 5% w/v sclerotia powder refluxed in a lye solution of initial pH 12.5 and for all reaction times, displayed only one major band, with a R_f_ value of 0.07 (intense purple) suggesting the degradation of less polar compounds to LSA and iso-LSA during treatment under these conditions. The ^1^H NMR spectra of the control samples (Fig. 4) and the samples with an initial lye pH of 10.5 and 11.5 in CDCl_3_ revealed several peaks at the low field chemical shift region at 9.0–10.0 ppm, corresponding to the proton of the amide linkage of the different ergopeptides connecting the lysergic acid structure and the tripeptide-based moiety (19-NH). A singlet at 7.9–8.1 ppm, assigned to the indole nitrogen proton (1-NH), aromatic overlapping multiplets at 7.1–7.6 ppm (H-12,-13,-14), overlapping singlets at 6.9 ppm corresponding to a proton at α-position to a pyrrolic nitrogen (H-2), overlapping vinylic signals at 6.38–6.43 ppm (H-9), and methyl singlets at 2.6–2.7 ppm (H-17) were consistent with the presence of the ergoline skeleton (Fig. 4). Ergokryptine (Ekr) and ergocristine (Ecr) were identified as the main ergopeptines in the control samples based on their NMR data available in the literature^38^, presented in the inset table of Fig. 4.

**Fig. 5:**
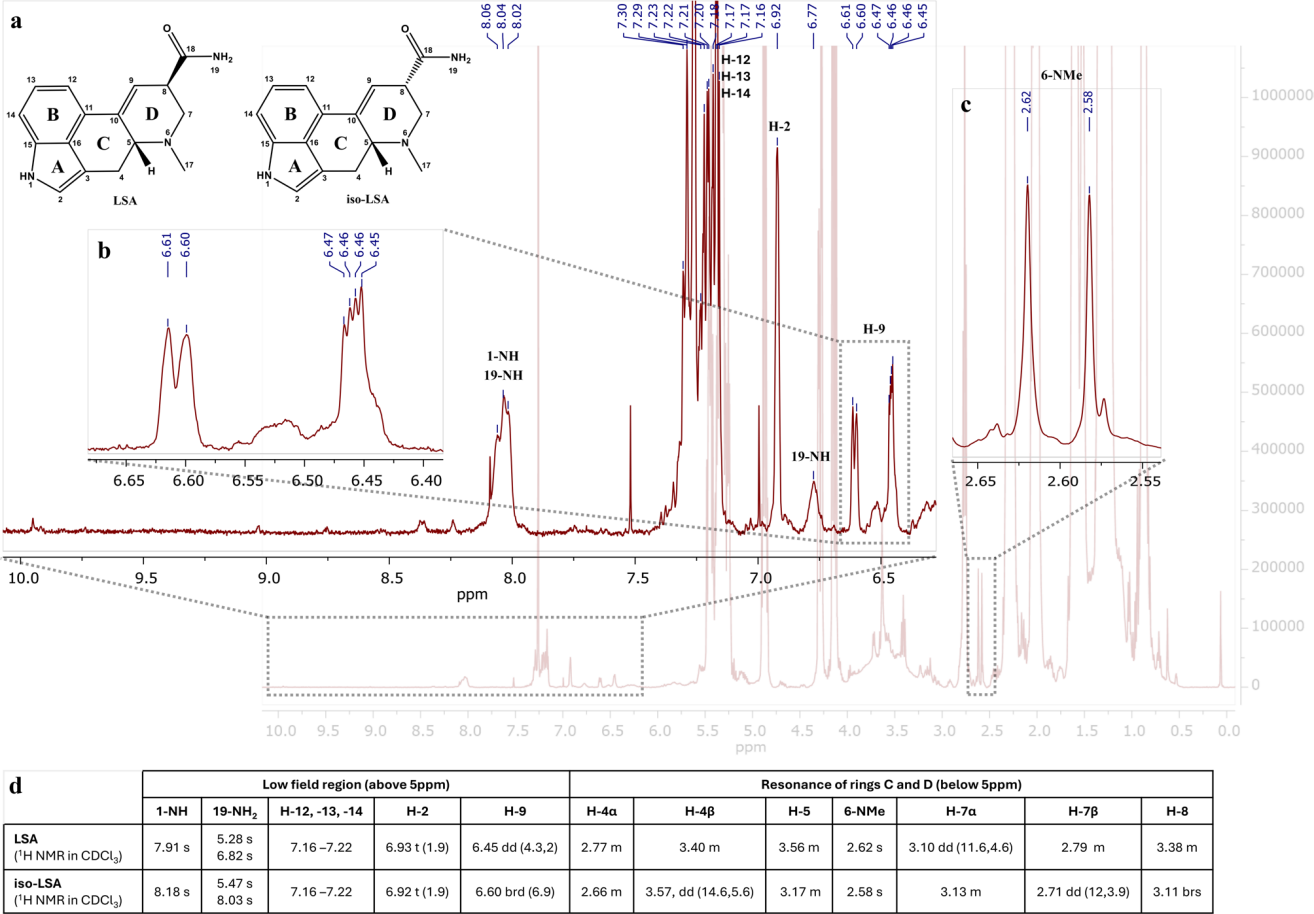
^1^H NMR spectrum in CDCl_3_ of the 5% w/v ergot test sample, refluxed in lye pH 12.5, 15 min. The spectrum confirms the hydrolysis of ergopeptides, evidenced by the disappearance of Ecr/Ekr amide signals and the emergence of characteristic LSA and iso-LSA peaks. Insets (a–c) highlight the diagnostic shifts in the low-field (19-NH, H-9) and high-field (6-NMe) regions. Inset table (d) summarizes the NMR assignments for low field region and rings C and D for LSA and iso-LSA in CDCl_3_ according to Table 1.

In contrast to the control samples, in the 5% w/v test samples of ergot refluxed in an initial lye solution of pH 12.5, across all reaction durations, the peaks downfield at 9.0–10.0 ppm corresponding to the amide protons of position 19 and the peaks within 7.0–7.6 ppm corresponding to the hydroxyls of the peptidic moiety of Ecr and Ekr (shown in Fig. 4), were absent, suggesting the cleavage of the peptidic bond between the lysergic acid structure and the tripeptide-based part (Fig. 5). Instead, two new singlets appeared at 6.8 and 8.0 ppm corresponding to one of the two 19-NH_2_ protons of LSA and its C-8 epimer, iso-LSA, the other two being overlapped by other peaks in the extract within 5.3–5.5 ppm. Additionally, the characteristic peaks for the vinylic hydrogen of the D ring (H-9) of LSA at 6.46 ppm and iso-LSA at 6.60 ppm were detected in the absence of the Ecr and Ekr peaks. Finally, the 6-NMe proton singlets of Ekr and Ecr, originally at 2.67 and 2.66 ppm, were replaced by the LSA and iso-LSA 6-NMe singlets at 2.62 and 2.58 ppm, respectively, further confirming the complete hydrolysis of both Ekr and Ecr, yielding LSA and iso-LSA (Fig. 5).

**Fig. 6:**
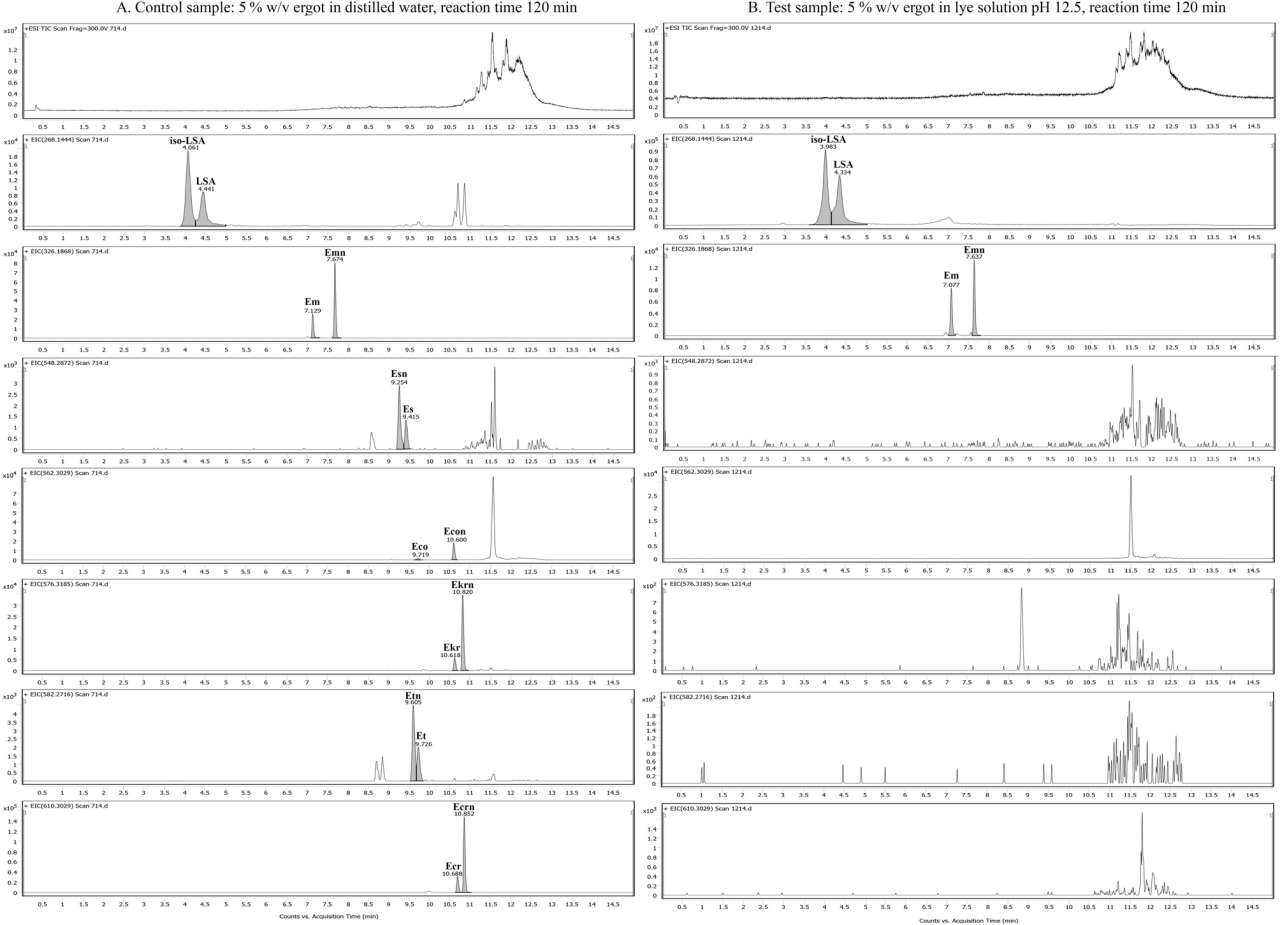
Qualitative MS analysis comparison. Column A: TIC and EICs of the control sample (5% w/v ergot in distilled water, 120 min), showing the presence of all predominant ergopeptides and their C-8 epimers. Column B: TIC and EICs of the test sample (5% w/v ergot in lye pH 12.5, 120 min), demonstrating the complete hydrolysis of ergopeptides and the resulting predominance of LSA and iso-LSA. Chromatographic conditions are detailed in Sect. 2.8; reference standard chromatograms are available in Supplementary Fig. S24.

The UHPLC/QTOF-HRMS qualitative analysis confirmed the degradation of ergopeptides and further indicated their hydrolysis to lysergamides, consistent with the NMR data. More specifically, out of the 36 test samples, in those prepared with 5% w/v ergot refluxed in an initial lye solution of pH 12.5 for 120 min, none of the predominant ergopeptides were detected, namely ergosine (Es), ergocornine (Eco), ergokryptine (Ekr), ergotamine (Et), and ergocristine (Ecr) neither their corresponding C-8 epimers, namely ergosinine (Esn), ergotaminine (Etn), ergokryptinine (Ekrn), ergocorninine (Econ) and ergocristinine (Ecrn). This suggests their complete degradation and hydrolyzation to LSA and iso-LSA, while the signals corresponding to ergometrine (Em) and ergometrinine (Emn) were observed (Fig. 6, column B). In contrast, in the control (Fig. 6, column A) and in all other test samples, all six predominant EAs, together with LSA and their related C-8, epimers were detected. Hydrolysis under these conditions was reproducibly observed across experiments performed on different days using independently prepared lye solutions.

## Quantitation of LSA and iso-LSA in putative kykeon preparations

After UHPLC/QTOF-HRMS quantitation of LSA and iso-LSA via standard addition calibration across the 48 experimental runs using varying reaction parameters, it was determined that LSA and iso-LSA can be produced at 0.54 mg g^− 1^ and 0.48 mg g^− 1^ of ergot used, respectively, while fully hydrolyzing the toxic ergopeptides, when refluxed for 120 min in an initial lye pH of 12.5 and at a concentration of 5% w/v ergot in lye. Higher levels of LSA and iso-LSA in mg g^− 1^ of ergot used were observed for reaction times of 15 to 60 min under the same conditions, and also when using a 10% w/v ergot concentration, and for all reaction durations under the same pH conditions (pH 12.5); however, toxic ergopeptides were still detectable in these extracts, indicating only partial hydrolysis. The highest LSA yield in mg g^− 1^ of ergot used was observed at an initial lye pH of 12.5 with 10% w/v ergot concentration after 60 min, reaching 0.81 mg g^− 1^; for iso-LSA a 1.12 mg g^− 1^ was observed at the same conditions after 30 min. In all other experiments conducted at initial lye pH values of 10.5 or 11.5, and across all reaction times and ergot concentrations tested, intermediate levels of LSA and iso-LSA were observed, while residual toxic ergopeptides were consistently detected. Regarding the maximum absolute amounts of LSA and iso-LSA produced per 10 mL aliquot, the highest observed yields were 1.15 mg of LSA obtained at 120 min, initial lye pH 12.5 and 20% w/v ergot concentration and 1.12 mg of iso-LSA, at 30 min, initial lye pH 12.5 and 10% w/v ergot concentration, yet residual ergopeptides were still detectable under these conditions as well. UHPLC/QTOF-HRMS quantitation results for LSA and iso-LSA together with dry extract yields and related calculations, are provided in the Supplementary Table S2.

### Modeling and evaluation of LSA and iso-LSA yield responses

**Fig. 7:**
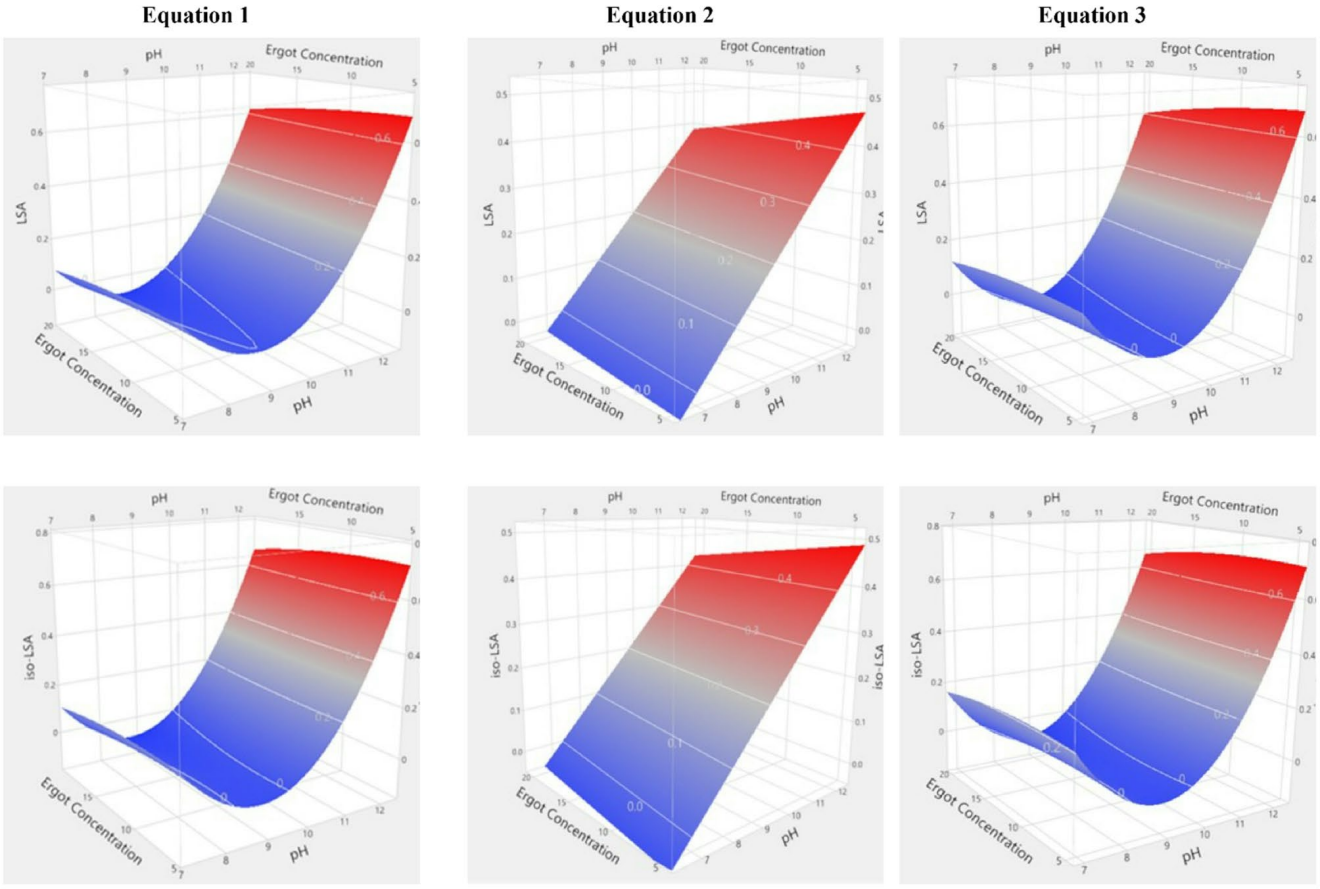
Surface plot based on the model equations applied for LSA and iso-LSA yield (mg g^− 1^ of ergot used) in the different “kykeon” preparations at reaction time T = 60 min.

**Fig. 8:**
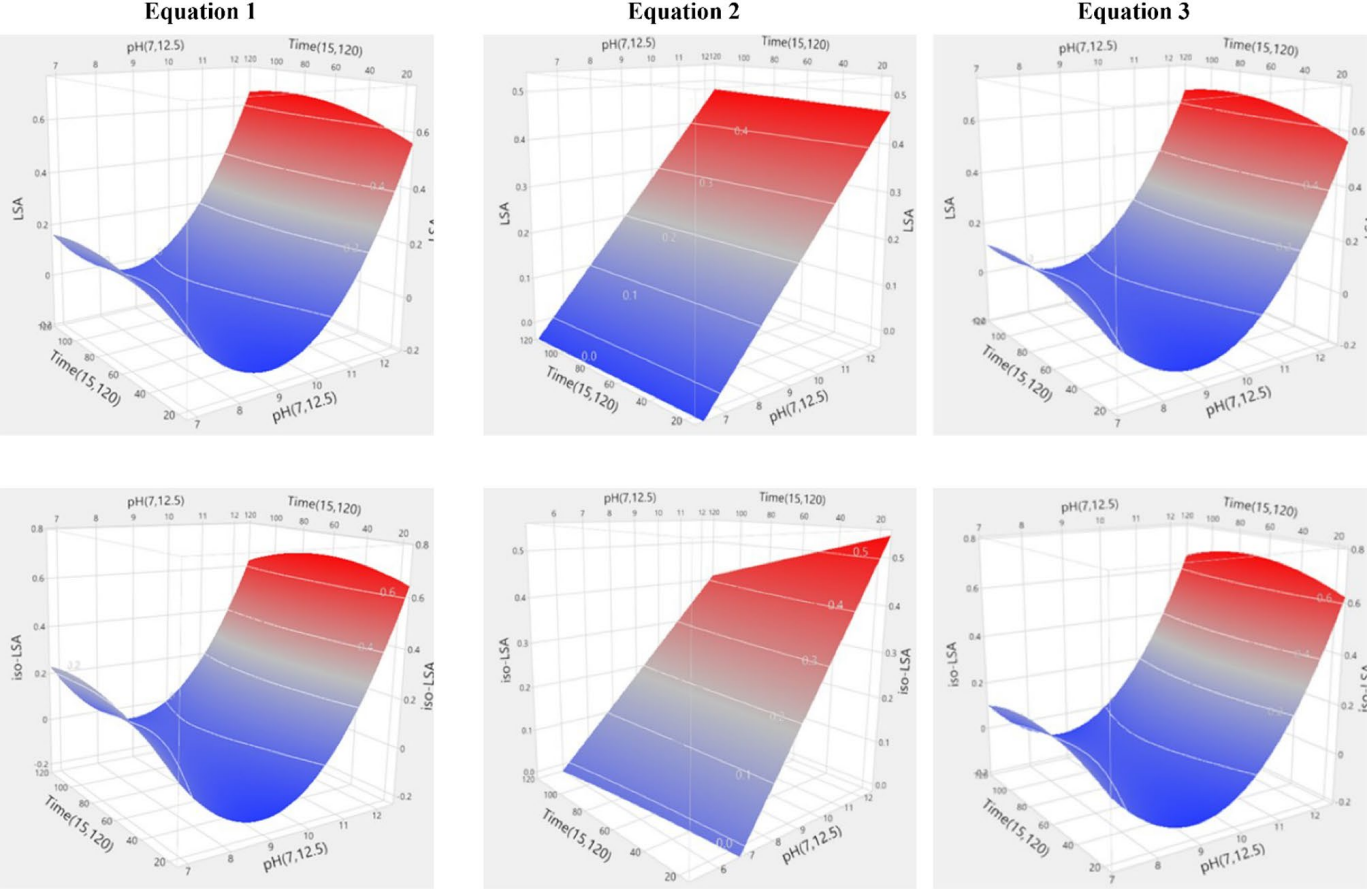
Surface plot based on the model equations applied for LSA and iso-LSA yield (mg g^− 1^ of ergot used) in the different “kykeon” preparations at ergot concentration in lye w/v = 10%.

ANOVA revealed that all models were highly significant (*p* < 0.0001). Lye pH was the sole significant predictor (*p* < 0.0001), with its quadratic term also significant in second-order models. No interactions among independent variables were detected (Supplementary Tables 3 and 4). The summary of fit statistics for the model equations applied to LSA and iso-LSA showed strong agreement between predicted and observed values. All models except Eq. 2 yielded high coefficients of determination (R^2^), indicating that most variability in the responses was explained by the fitted equations. Incorporating a squared term for the independent variable further increased explanatory power (Eq. 1: R^2^ = 0.8300, Eq. 3: R^2^ = 0.8217 for LSA; Eq. 1: R^2^ = 0.7816, Eq. 3: R^2^ = 0.7616 for iso-LSA). The adjusted coefficients of determination (R^2^adj) were similarly high and closely matched the R^2^ values (Eq. 1: R^2^adj = 0.7898, Eq. 3: R^2^adj = 0.7956 for LSA; Eq. 1: R^2^adj = 0.7299, Eq. 3: R^2^adj = 0.7267 for iso-LSA), confirming model robustness while accounting for the number of predictors. In contrast, the Eq. 2 model produced the lowest coefficients (R^2^ = 0.4964, R^2^adj = 0.4228 for LSA; R^2^ = 0.4215, R^2^adj = 0.3369 for iso-LSA). The corresponding root mean square errors (RMSE) are reported in Supplementary Table S5. Overall, these findings indicate that models incorporating a squared term of the independent variable provide greater predictive accuracy for both analytes. The simplest statistically significant equations (*p* < 0.0001) for LSA and iso-LSA were those using pH as the independent variable in first- and second-order forms (R^2^ = 0.7824, R^2^adj = 0.7726, RMSE = 0.2674 for LSA; R^2^ = 0.7390, R^2^adj = 0.7274, RMSE = 0.1457 for iso-LSA). Indicative three-dimensional response surface plots are shown in Figs. 7 and 8.

## Discussion

NMR and UHPLC/Q-TOF-HRMS analysis of 48 experiments employing varying reaction parameters indicated that LSA and iso-LSA can be generated through complete degradation of toxic ergopeptides using a simple lye treatment. This transformation occurs at 5% w/v ergot sclerotia in an initial pH 12.5 lye solution and reacting for at least 120 min. The proposed mechanism for the hydrolysis and chemical transformation of ergopeptines Erk and Erc into LSA and iso-LSA is presented in the Supplementary Fig. S25.

The analysis focused on targeted EAs, including those monitored under EU directives and EFSA recommendations, specifically covering the six main EAs (ergometrine (Em), ergosine (Es), ergocornine (Eco), ergokryptine (Ekr), ergotamine (Et), and ergocristine (Ecr)) and their related epimers (ergometrinine (Emn), ergosinine (Esn), ergotaminine (Etn), ergokryptinine (Ekrn), ergocorninine (Econ) and ergocristinine (Ecrn))^39^. Expected alkaline hydrolysis products (i.e., LSA and iso-LSA)^27,28^ were monitored, and full-scan UHPLC/Q-TOF-HRMS data were inspected for potential non-target degradation products. No abundant unexpected ergot-derived species with high-intensity signals corresponding to known toxicologically relevant compounds were detected above background levels. These observations were consistent with qualitative NMR analysis, which did not indicate the presence of additional major alkaloid species. The persistence of trace amounts of Em and Emn does not compromise the safety of the preparation. Unlike the complex ergopeptides, which were completely hydrolyzed and are responsible for the gangrenous effects of ergotism, Em and Emn are simple lysergamides with a well-characterized medicinal profile and significantly lower peripheral vasoconstrictive toxicity^25^. In the context of the Eleusinian Mysteries, detoxification primarily entails the elimination of lethal ergopeptides, a requirement successfully met by the lye-based treatment.

In recent years, the debate over the use of entheogens in the Eleusinian Mysteries has remained active, with scholars split between enthusiastic proponents and uncompromising skeptics^6^. Among the former, another proposal suggests that an acidic barley-water mixture may convert toxic EAs into iso-LSH, the C-8 epimer of LSH (Fig. 1), with a subsequent alcohol extraction recovering these compounds^10^. However, alcohol is not part of the kykeon, LSH and iso-LSH are extremely unstable, thus readily degrading into acetaldehyde and the corresponding amides, merely serving as prodrugs of LSA and iso-LSA^40^. Regarding the latter group, many classicists remain skeptical of the “psychedelic Eleusis” hypothesis, not by presenting new disproof, but by warning against reliance on speculative evidence and noting the absence of ergot residues in Eleusinian vessels^11^. This absence, however, is not decisive, since no systematic research has ever been conducted or since permission for such has never been granted. Concrete findings however do exist elsewhere (see the ergot finding from Mas Castellar^23,24^, which are left unmentioned, even though they lend empirical weight to the hypothesis, demonstrating that ergot was, in fact, connected with the worship of the Eleusinian Goddesses. Many critics also frame a false dilemma: ergot equals ergotism^11^. Yet the history of medicine shows otherwise; crude ergot was used in obstetrics for centuries, and innumerous isolated and semi-synthesized EAs were and are still in use in clinical practice^25^. The key is dose and processing, as demonstrated in this study, not inevitability of poisoning.

Regarding the psychoactivity of LSA and iso-LSA, it is well documented in both ethnobotanical^41–43^ and pharmacological literature^44–47^, being also the main active compounds in *ololiuqui* (“round things”), an entheogenic preparation of *Ipomoea corymbosa* seeds used by the Aztecs^43^. Although pure LSA shows lower receptor binding affinities than LSD in vitro^44^, in a recent review, it was demonstrated that among its reported effects are euphoria and hallucinations^45^. Consistently, in a β-arrestin2 recruitment assay, LSA exhibited approximately 100-fold lower potency than LSD at both the 5-HT_2_A and 5-HT_2_B receptors, while remaining notably active, with EC_50_ values of 58 nM and 54 nM, respectively^46^. Regarding iso-LSA, it has demonstrated measurable affinity for central 5-HT binding, as reported in early QSAR studies^47^. Notably, it displaced both ^3^Hserotonin and ^3^HLSD from rat cerebral cortex membranes with pIC50 values of 7.00 and 6.70, respectively and exhibits a log P of 0.95, consistent with moderate lipophilicity and potential for blood-brain barrier permeability^47^. In vivo studies further support its central activity^48^. Iso-LSA administered intraperitoneally to rats reached measurable concentrations in the brain correlating with behavioral effects such as decreased conditioned avoidance response, indicating that the parent compound, rather than a metabolite, acts as the psychoactive agent^48^. Therefore, despite its isomeric difference from LSA, iso-LSA (being the C-8 (*S*) epimer, which are usually considered inactive)^39^ may retain central serotonergic activity relevant to psychedelic potential. Additionally, the result of each of the kykeon preparations was an equilibrium mixture of LSA and iso-LSA, which may be more psychoactive than either pure alkaloid alone^28^. However, further pharmacological and toxicological evaluations are necessary to define the exact safety profile and receptor affinities of the resulting mixtures. Another key factor is that “set and setting” predominantly shape the experience, the substances merely acting as tools^2^. In an ancient ritual framework, marked by botanical synergism, fasting, and heightened expectancy, their psychedelic potential would have been amplified. At Eleusis, optimal ceremonial conditions gave these entheogens a meaningful role, showing that their effects must be understood within a religious context, rather than purely pharmacological terms.

One concern regarding the lye hypothesis for the detoxification of the ergopeptides in the kykeon is the potability of the resulting preparation; a solution with a pH > 11.5 is highly caustic for oral consumption and can injure esophageal epithelium^49^. However, it was demonstrated that an initial highly basic pH value can be neutralized by exposure to atmospheric CO_2_ and sclerotia addition, while maintaining reaction performance. The addition of the resulting solution to the barley-mint preparation, typically slightly acidic, would further neutralize the pH, rendering kykeon safe to drink.

With regard to origin, neither the geographic source nor the precise ergopeptides composition of *C. purpurea* sclerotia is decisive. Phylogenetic and comparative genomic analyses indicate that ergopeptide biosynthesis emerged in the *C. purpurea* lineage after the genus diversified in the Paleocene-Eocene (~ 56–34 Mya), through recruitment of *lpsA/lpsB* nonribosomal peptide synthetases into the ancestral EA core pathway^50–52^. This evolutionary timeframe addresses Hofmann’s concern (“*We have no way to tell what the chemistry was of the ergot of barley or wheat raised on the Rarian plain in the 2nd millennium B.C.*”)^8^. Considering the cosmopolitan distribution of *C. purpurea* (following Pooideae)^36^ and the evolutionary scale, two millennia represent a negligible interval; thus, the ergot infecting ancient Eleusinian barley likely contained the same major ergopeptide classes as modern strains. Even if minor compositional differences had existed, all ergopeptides ultimately hydrolyze into the same lysergamides, accompanied only by harmless amino acid by-products (Supplementary Fig. S25). Another fact to note is that the *Claviceps paspali* theory has been dismissed, as its host plants are non-native to Europe and only became naturalized in the 19th century^53^, excluding their presence in ancient Greece.

Regarding the sourcing of ergot by the priestesses, one potential conduit lay in the cultic grain offerings. Every city-state contributed cereals to Eleusis, a practice rooted in a famine-era decree from the Delphic oracle^3^. Under Athens, the Mysteries became Panhellenic, with heralds summoning poleis required to dedicate first harvest of barley before public use^3^. These offerings to Demeter could have introduced ergot-contaminated grains into the sanctuary, if not collected directly by the priestesses from the Rarian field. The fertile Thriasian plain, Eleusis’ granary, with its heavy soils and marshy patches^54^, offered suitable microhabitats for *C. purpurea*. Paleoenvironmental and hydrogeological data from Eleusis Bay^55^ and across the Attica peninsula indicate stable Mediterranean conditions (dry summers, humid winters)^56^ with locally waterlogged areas in Classical antiquity^54^. Such conditions would have limited the extent of infection and precluded epidemic ergotism of the kind seen in medieval Northern Europe. However, localized contamination could nonetheless have sufficed to supply the small but controlled quantities required for ritual use within the sanctuary.

Regarding the quantities of ergot required, the average content of EAs in sclerotia of *C. purpurea sensu lato* is a few milligrams per gram (typically ranging from 0.01 to 1.3 mg g^− 1^ to 2.88–7.26 mg g^− 1^)^57^. Τhe minimum effective dose of pure LSA is approximately 0.5 mg^43^, and up to two thousand participants could have attended the Eleusinian initiations simultaneously^3^. Consequently, the priestesses of the temple would likely have required few kilograms of sclerotia, along with twenty times that amount in freshly prepared lye solution. These quantities could have been readily obtained and processed within the secrecy of the temple. Since our results showed that 0.54 and 0.48 mg g^− 1^ of ergot yield LSA and iso-LSA, respectively, under optimal conditions (5% w/v ergot in lye, pH 12.5, 120 min at 100 °C), about 1 g of ergot would suffice for each initiate. The required amount might have been even less, given the nine-day fasting period observed by the initiates before the consumption of the kykeon and the entheogenic intensification in a ritual setting, as mentioned above.

Finally, focusing on elements of the kykeon mixture other than the hypothesized ergot and beyond what was outlined in the introduction, a potential synergistic role of pennyroyal’s constituents is proposed. The pennyroyal essential oil’s principal monoterpene, (*R*)-(+)-pulegone, is known to interact with CNS pathways involved in nociception, sedation, psychotropic activity, and analgesia^58,59^. Additionally, its aqueous extract, rich in rosmarinic acid, modulates MAO-A, suggesting nootropic and mood-stabilizing effects^60^. Therefore, it is proposed that these secondary metabolites may contribute to a psychostimulant-synergistic “entourage effect” with LSA and iso-LSA, analogous to the terpenes–THC synergy in cannabis^61^ and the DMT–β-carbolines in ayahuasca^62^, possibly reducing anxiety and prolonging the entheogenic effects. This hypothesis remains to be validated through targeted co-administration studies.

## Conclusion

Our findings demonstrate that toxic ergopeptides can be chemically transformed into psychoactive substances through an ancient process involving reaction in lye, a technique that could have been employed by the ceremonial priestesses of Eleusis. These results support the hypothesis that the entheogenic properties of kykeon could be attributed to the use of ergot, as suggested by R. Gordon Wasson, Albert Hofmann and Carl A. P. Ruck almost half a century ago. It should be noted that the purpose of this work was not to establish a novel analytical method for the quantitation of LSA and iso-LSA, nor to optimize the hydrolysis reaction conditions through response surface methodology, nor to re-evaluate the pharmacological properties of EAs, LSA and iso-LSA, which are already well documented. Rather, the focus of the study was to demonstrate the feasibility of this transformation in the specific historical-technological context under investigation, which has been successfully achieved. Looking forward, it is anticipated that the application of advanced organic residue analysis to existing and future archaeological discoveries from the site of Eleusis may provide the necessary material evidence to further substantiate and enrich the “psychedelic Eleusis” hypothesis.

### Methods

#### Source, micromorphological and molecular identification of ergot

Small quantities of identified ergot sclerotia of *Claviceps purpurea* (Fr.) Tul. were generously provided by the herbarium collection of the Plant Pathology Laboratory, Faculty of Agriculture, Aristotle University of Thessaloniki, Thessaloniki, Greece (October 2024), and by the Culture Collection of Clavicipitaceae of the Institute of Microbiology, Academy of Sciences of the Czech Republic, Prague, Czech Republic (January 2025). Additionally, for the purposes of our experiments, larger quantities of ergot sclerotia were either collected directly from private fields or obtained from the waste fraction at a grain elevator cleaning facility in Alberta, Canada (September 2024), and were kindly provided to us by the respective landowners or facility operators. The identification of the fungal material was verified by Z.G.-Z. after systematically studying the culture derived from it (morphological and molecular characterization). Both sclerotia and the strain are safeguarded at the Mycetotheca ATHUM (accessed as ATHUM10382). All sclerotia were kept at room temperature (20–25 °C) in a dry, dark environment until analysis. To identify the field-collected sclerotia, the following procedure was employed. The sclerotia were surface sterilized in 70% ethanol for 10 s followed by 1 min in 10% bleach and rinsed several times with sterile distilled water. After air drying on sterile gauzes in an Airstream Class II biohazard safety cabinet (ESCO Micro Pte. Ltd., Singapore), the sclerotia were aseptically cut into 5 mm pieces and plated by three-point inoculation on PDA (39 g/L of distilled water, Becton, Dickinson & Co., Franklin Lakes, NJ, USA) in pre-sterile polystyrene Petri plates, 92 mm in diameter and 16 mm high with ventilation cams (Sarstedt AG & Co. KG, Nümbrecht, Germany). The plates were incubated at 23 °C for 14 days in darkness in a FTC90E refrigerated incubator (Velp Scientifica Srl., Milano, Italy).

For the micromorphological characterization of the strain, sterile distilled water was poured over the surface of the colonized PDA to create a conidial suspension, which was then used to prepare a microscope slide. Images of the conidia at 400× magnification were captured using Differential Interference Contrast (DIC) optics on an Axio Imager.A1 microscope equipped with an AxioCam MRc camera (Carl Zeiss Microscopy GmbH., Jena, Germany). Genomic DNA was isolated from 100 mg of fungal tissue using the HigherPurity Plant DNA Purification Kit (Canvax Biotech, Spain), following the manufacturer’s instructions. DNA integrity was evaluated via 0.8% agarose gel electrophoresis, while purity and concentration were assessed using a NanoDrop spectrophotometer and a Qubit fluorometer, respectively. Amplification of the fungal internal transcribed spacer (ITS) region was performed using the ITS1 (5′-GTCCCTGCCCTTTGTA-3′) and ITS2 (5′-CCTGGTGGTTTCTTTTCC-3′). The reaction conditions followed a previously published protocol^63^.

### Preparation of wood ash derived lye solutions

In order to obtain the lye solution, mixed oak and olive tree dry wood chips (proportions of approximately 0.55 kg), supplied from a local lumberyard in Athens, were incinerated at 600 °C in a NABER model L 51/S ashing furnace (Nabertherm GmbH., Lilienthal, Germany) under a fume hood for three h to produce wood ash (approximately 15 g per 0.55 kg of starting material). The lye solution was prepared by boiling one part of wood ash into two parts of distilled water in a glass beaker for ten min on a MSH-20D magnetic stirrer with hotplate (Witeg Labortechnik GmbH., Wertheim, German), distilled water added to compensate for water loss and then allowed to sit overnight. After the settling period, the supernatant was decanted and filtered with filter paper via a Büchner funnel. The filtrate was collected, being the primary lye solution and its pH value was measured at 12.5. The whole process was repeated to prepare additional solutions as needed. Lye solutions with pH values of 10.5 and 11.5 were prepared by exposing aliquots of the primary lye solution in glass beakers to atmospheric air for controlled durations (2 and 5 days respectively). Distilled water was added as needed to compensate for evaporation losses. The term “initial pH” refers to the alkalinity of the lye solutions before the addition of ergot sclerotia.

### Design and analysis of alkaline hydrolysis experiments

Alkaline hydrolysis experiments were conducted on separate experimental days using freshly prepared lye solutions. Independent lye preparations were generated from separate wood ash batches following the same protocol described above, and their initial pH values were verified prior to use before each set of experiments to ensure inter-batch reproducibility. To optimize the hydrolysis conditions of the EAs, a full factorial design with varying levels was employed. Three quantitative variables were selected: initial lye pH (10.5, 11.5, 12.5), ergot powder concentration in the lye (5%, 10%, 20% w/v), and reaction time (15, 30, 60, 120 min) at 100 °C. Towards this end, ergot sclerotia of the field-collected stain were pulverized using a mortar and pestle. The resulting powder was then boiled under reflux in the lye solutions and in distilled water (neutral pH), the latter serving as control samples. This design comprised a total of 48 experimental runs. For each experiment, a 45 mL sample for each concentration of pulverized ergot powder in the respective lye solution or in the distilled water was prepared in a 100 mL flask and heated in the stirring oil bath at 100 °C and 200 rpm under reflux on the magnetic stirrer’s hotplate. For each treatment, 10 mL aliquots were collected at the defined intervals, measured from the onset of boiling. Each aliquot was then put aside to reach room temperature (25 °C), its pH was measured and made basic to pH 9.5, if lower, with the addition of sodium bicarbonate (ReagentPlus, ≥ 99.5% purity, Sigma-Aldrich, Merck KGaA, Darmstadt, Germany) and was exhaustively extracted trice with 30 mL of ethyl acetate and trice with 30 mL of chloroform (both Analytical reagent grade, Thermo Fisher Scientific Inc., Waltham, MA, USA). The combined organic phases for each extract were collected, dried over anhydrous sodium sulphate (Merck KGaA, Darmstadt, Germany), evaporated under reduced pressure via a R210 rotavapor equipped with a V700 vacuum pump (BÜCHI Labortechnik AG, Flawil, Switzerland) at 35 °C, weighed, and stored at − 20 °C under a nitrogen atmosphere until analysis.

### Isolation of LSA and iso-LSA from *Argyreia nervosa *seeds

Due to the lack of comprehensive NMR data for LSA and iso-LSA in the scientific literature, the substances were isolated following a series of acid-base extractions and crystallizations from *Argyreia nervosa* seeds (supplied by Ethos Herbals, Athens, Greece), which like *ololiuqui* contain these metabolites but at higher concentrations, exhibiting the highest indole alkaloid content (0.5–0.9%) reported among Convolvulaceae species^64^. For this purpose, a crude alkaloid extract was prepared from 10 g of seeds. The seeds were initially pulverized with a mortar and pestle and defatted with 200 mL of petroleum ether (boiling point 40–60 °C, Certified ACS grade, Thermo Fisher Scientific Inc., Waltham, MA, USA) in a round-bottom flask heated in the oil bath set at 35 °C and 200 rpm under reflux on the magnetic stirrer’s hotplate for 30 min, transferred in 50 mL falcon tubes, centrifuged at 4000 rpm for 5 min in a Frontier 5816 centrifuge (OHAUS Corporation, Parsippany, NJ, USA), the supernatants discarded, the pellets dried in a fume hood, transferred into a 250 mL Erlenmeyer flask and wetted with 50 mL of 10% aqueous ammonium hydroxide solution (Thermo Fisher Scientific Inc., Waltham, MA, USA). Then 100 mL of diethyl ether (Emplura series, Merk KGaA., Gernsheim, Germany) were added, and the mixture was shaken at 200 rpm on a STUART SSL1 orbital shaker (Cole-Parmer Instrument Company LLC, Vernon Hills, IL, USA) for 30 min. The ether layer was collected by decantation, and the residue was extracted twice more, each time with 100 mL of diethyl ether. The combined organic phases were extracted with 3 × 100 mL of 0.1 N aqueous H_2_SO_4_ (Merck KGaA, Darmstadt, Germany). The acidic aqueous phase was collected, adjusted to pH 10 with 25% aqueous ammonia solution (Emsure series, Merck KGaA, Darmstadt, Germany), and extracted with 3 × 100 mL of chloroform. The combined organic phases were collected, dried over anhydrous sodium sulphate and evaporated under reduced pressure via the rotavapor at 35 °C. The compounds were subsequently crystallized according to the scheme detailed in a previously published protocol^64^, were recrystallized from methanol, and stored at − 20 °C under a nitrogen atmosphere until analysis. Their ^1^H NMR spectra in DMSO-d_6_ were compared with those provided in the CoAs of reference standard materials (methylergometrine EP impurity C, catalog #M-130008, ^1^H NMR in DMSO-d_6_ spectrum number 6139-006A8 and methylergometrine EP impurity E, catalog #M-130010, ^1^H NMR in DMSO-d_6_ spectrum number 6139-006A7, for LSA and iso-LSA respectively, TLC Pharmaceutical Standards Ltd., Newmarket, ON, Canada).

### Thin layer chromatography screening

Samples of the kykeon’s extracts were primarily monitored with TLC with a chloroform/methanol (95:5 v/v) system (both Analytical reagent grade, Thermo Fisher Scientific Inc., Waltham, MA, USA) on 60 F254 silica gel 200 μm thickness aluminum sheets (Merck KGaA, Darmstadt, Germany), observed under UV light at 254 nm and 366 nm and visualized with Ehrlich’s reagent [1 g of p-dimethylaminobenzaldehyde (p-DMAB, 98% purity, Merck KGaA (Sigma-Aldrich), Darmstadt, Germany) in 50 mL of ethanol and 50 mL of concentrated hydrochloric acid (aqueous HCl 37%, Emsure series, Merck KGaA, Darmstadt, Germany)].

### Nuclear magnetic resonance spectroscopy

NMR spectra of isolated LSA and iso-LSA were recorded in chloroform-d (CDCl_3_, 99.8% atom D, Sigma-Aldrich, Merck KGaA, Darmstadt, Germany) and dimethyl sulfoxide-d_6_ (DMSO-d_6_, (CD_3_)_3_SO, 99.5% atom D, Sigma-Aldrich, Merck KGaA, Darmstadt, Germany). Samples of the dry kykeon’s extracts were redissolved in CDCl_3_ and monitored with ^1^H-NMR. All NMR experiments were performed at 298 K on a Bruker AVANCE III 400 MHz instrument (Bruker Corporation, Billerica, MA, USA) equipped with a BBI 5 mm probe. The NMR chemical shifts are given on the *δ* scale in ppm taking as reference the residual solvent peaks at *δ* 7.26 for CDCl_3_ and *δ* 2.50 for DMSO-d_6_, while integration, multiplicity, and coupling constants (*J* in Hz) are reported along with the signals.

### Sample preparation for UHPLC/Q-TOF-HRMS

For UHPLC/Q-TOF-HRMS quantitation of LSA and iso-LSA, standard addition calibrations were performed, while the major EAs (ergometrine (Em), ergosine (Es), ergocornine (Eco), ergokryptine (Ekr), ergotamine (Et), and ergocristine (Ecr)) and their C-8 epimers (ergometrinine (Emn), ergosinine (Esn), ergotaminine (Etn), ergokryptinine (Ekrn), ergocorninine (Econ) and ergocristinine (Ecrn)), were qualitatively identified in the samples by comparison of retention times and mass spectra with those of reference standards. A mixed solution (0.1 µg mL^-^^1^ each in acetonitrile) was prepared as described in a previously published protocol^65^. LSA and iso-LSA standards were purchased from TLC Pharmaceutical Standards Ltd. (Newmarket, ON, Canada), fine film dried standards of Em, Emn, Et and Etn were purchased from Romer Labs (Getzersdorf, Austria), while the remaining standards (Es, Esn, Eco, Econ, Ekr and Ekrn) were obtained from Techno Spec (Barcelona, Spain).

Samples of the dry kykeon extracts were redissolved in water/methanol (5:95 v/v), both LC-MS grade (water from Merck KGaA (Sigma-Aldrich), Darmstadt, Germany; methanol from Avantor (VWR International), Radnor, PA, USA), at a concentration of 40 µg mL of dry extract in the water/methanol mixture for controls and samples in lye at pH 10.5, and at a concentration of 20 µg mL of dry extract in the water/methanol mixture for samples in lye at pH 11.5 and 12.5. Each sample set included a non-fortified sample and four fortified samples at concentration levels of 25, 50, 75, and 100 ng mL for each analyte, to compensate for matrix effects and establish the standard addition calibration curves. All samples were processed in duplicate and filtered using PTFE syringe filters (0.22 μm, 13 mm; Clarify, Phenomenex, Torrance, CA, USA) prior to injection. The peak areas were plotted as a function of the added concentrations of each analyte.

### Chromatographic conditions

Chromatographic analysis was carried out based on a previously in-house developed method for EAs analysis^65^, implemented on an Agilent 1290 Infinity II system, composed of a Multicolumn Thermostat (G7116B), Vialsampler (G7129B), Flexible Pump (G7104A), and an Agilent 1200 Series Micro Degasser (G1379B), (Agilent Technologies, Santa Clara, CA, USA) interfaced to the MS. The column used for the analysis was a ZORBAX Eclipse Plus C18 Rapid Resolution HD 50 × 2.1 mm, 1.8 μm (Agilent Technologies, Santa Clara, CA, USA), thermostated at 35 °C, the mobile phase flow rate was set at 0.4 mL min^-^^1^ and the injection volume at 5 µL. Mobile phase consisted of water (solvent A) and MeOH (solvent B) both acidified with 0.3% v/v of formic acid (Formic acid ≥ 99%, HiPerSolv for LC-MS, VWR Chemicals, Radnor, PA, USA). The gradient was conducted as follows: 0 min, 5% B; 4.7 min, 5% B; 6.5 min, 41.5% B; 9 min, 44.5% B; 10 min, 95%; 11 min, 95% B; 12 min, 5% B. The gradient was set at 5% B for the last 3 min to re-equilibrate the column before the next injection, accounting for a total run time of 15 min. In order to minimize epimerization, the vial sampler was thermostated at 10 °C during analysis, while the injection sample sequence was limited to 12 h. Moreover, control standard solutions (mixed solution, 0.05 µg mL^-^^1^ of each analyte in water/methanol (5:95 v/v)) were injected at the beginning, middle, and end of each analysis sequence. A retention time window of ± 0.2 min was applied for compound identification.

### Mass spectrometry conditions

The mass spectrometer coupled with the UHPLC system was an Agilent 6550 iFunnel Q-TOF, equipped with a Dual AJS Electrospray Ionization (ESI) source (Agilent Technologies, Santa Clara, CA, USA), and operated in positive ionization mode [ESI(+)]. Non-fortified and fortified samples were analyzed using the All-Ions fragmentation acquisition mode. High-resolution accurate mass data were acquired with a QTOF HRMS method consisting of two sequential experiments at alternating collision energies: one full-scan at 0 V, followed by one All-Ions fragmentation scan at 30 V. The low-energy spectra (0 V) were used to obtain precursor ions, while the high-energy spectra (30 V) were used primarily to obtain fragment ions. The parameters of the Dual AJS ESI source were as follows: nebulizer gas (N_2_) pressure was set to 35 psig, whereas the drying gas (N_2_) flow rate was set to 15 L min^-^^1^ at 160 °C, and the sheath gas flow rate was established at 10 L min^-^^1^ at 260 °C. The capillary, nozzle, fragmentor and 1 RF Vpp octopole voltages were set at 3500, 600, 300 and 750 V, respectively. The instrument was calibrated and tuned according to procedures recommended by the manufacturer. HRMS and fragmentation data were stored in positive polarity using centroid mode at a scan rate of 10 spectra/s. Accurate mass spectra were acquired in the MS range 50–1000 *m/z*. To ensure the desired mass accuracy of recorded ions, continuous internal calibration was performed during analyses by using as reference mass the signals at 121.0509 *m/z* (protonated purine) and 922.0098 *m/z* (protonated hexakis(1 H, 1 H, 3 H-tetrafluoropropoxy)phosphazine; HP-0921) in the positive ionization mode (API-TOF Reference Mass Solution Kit, Agilent Technologies, Santa Clara, CA, USA). The reference nebulizer pressure for the internal calibrant was set to 0 psig. A mass error tolerance of ± 10 ppm was applied for compound identification.

### Data analysis

The NMR spectra were processed using TopSpin (version 4.4.1, Bruker BioSpin GmbH, Rheinstetten, Germany) and Mnova (version 15.1, Mestrelab Research S.L., Santiago de Compostela, Spain) software.

Precursor mass calculations and data processing for the UHPLC/Q-TOF-HRMS spectra and chromatograms were performed using Agilent MassHunter Workstation Software (version 10.0, Agilent Technologies, Santa Clara, CA, USA).

The chemical structures and figures were designed using ChemDraw software (version 23.1.1, Revvity Signals Software, Waltham, MA, USA).

Design of experiments, data processing, and evaluation of the effects of the three quantitative independent variables, i.e. lye pH (X1), ergot powder concentration in the lye in % w/v (X2), and reaction time in minutes (X3), on the production of the response variables, i.e. LSA and iso-LSA per mg g^-^^1^ of ergot used, were analyzed and plotted by JMP Pro software (version 17.1, SAS Institute Inc., Cary, NC, USA). Analysis of Variance (ANOVA) was performed at a significance level *p* ≤ 0.01 to evaluate the significance of individual process factors and their interactions on the basis of their F-and p-values.

The experimental data were first fitted in accordance to Eq. (1) as a second-order polynomial equation including the linear and interaction effects of each variable:

1 $$\:Y={\beta\:}_{0}+\:\sum\:_{j=1}^{k}{\beta\:}_{j}{X}_{j}+{\sum\:}_{j=1}^{k}{\beta\:}_{jj}{X}_{j}^{2}+\:\sum\:\sum\:_{i<j}{\beta\:}_{ij}{X}_{i}{X}_{j}\:\:$$

Then, the statistically insignificant terms were eliminated from the derived quadratic equation, leading to simpler equations, such as the two-factor-interaction (2FI) model, Eq. (2):

2 $$\:Y={\beta\:}_{0}+\sum\:_{j=1}^{k}{\beta\:}_{j}{X}_{j}+\sum\:\sum\:_{i<j}{\beta\:}_{ij}{X}_{i}{X}_{j}$$

or Eq. (3):

3 $$\:{Y=\beta\:}_{0}+\:\sum\:_{j=1}^{k}{\beta\:}_{j}{X}_{j}+\sum\:_{j=1}^{k}{\beta\:}_{jj}{X}_{j}^{2}\:$$

where Y = the predicted response, *β*_*0*_ = the constant term, *β*_*i*_ = the linear effect, *β*_*jj*_ = the squared effect and *β*_*ij*_ = interaction effect.

Subsequently, the statistically insignificant terms were eliminated from the derived equations, leading to a simpler equation.

## Supplementary Information

Below is the link to the electronic supplementary material.

Supplementary Material 1

## Data Availability

All data generated or analyzed during this study are included in this published article and its supplementary information files.
