# Supplementary material for: Investigating the psychedelic hypothesis of kykeon, the sacred elixir of the Eleusinian Mysteries
Source: Sci Rep. 2026 Feb 13;16:8757. doi: 10.1038/s41598-026-39568-3 (PMC12982482; doi:10.1038/s41598-026-39568-3)
Supplement: Supplementary file 1 — Supplementary Material 1 [file 41598_2026_39568_MOESM1_ESM.pdf]

Supplementary Data

Investigating the psychedelic hypothesis of *kykeon*, the sacred elixir of the Eleusinian Mysteries

Romanos K. Antonopoulos[1,2,+], Evangelos Dadiotis[1,2,+,*], Kostas Ioannidis[3], Antigoni Cheilari[1], Vangelis Mitsis[2], Ana M. Garcia-Campaña[4], Laura Gámiz-Gracia[4], Maykel Hernández-Mesa[4], Alfonso Narváez[4], Mark A. Hoffman[5], Carl A. P. Ruck[6], Zacharoula Gonou-Zagou[7], Nektarios Aligiannis[1], Prokopios Magiatis[1,*]

[1] *Laboratory of Pharmacognosy and Chemistry of Natural Products, Department of Pharmacy, National and Kapodistrian University of Athens (NKUA), Panepistimioupoli Zografou, 15771 Athens, Greece*
[2] *Hellenic Center for Entheogenic Research, NPC, Porto Rafti, 19003 Attica, Greece*
[3] *Laboratory of Sylviculture, Forest Genetics and Biotechnology, Institute of Mediterranean and Forest Ecosystems, Hellenic Agricultural Organization "Demeter", Ilissia, 11528 Athens, Greece*
[4] *Department of Analytical Chemistry, Faculty of Sciences, University of Granada, 18071 Granada, Spain*
[5] *The Wasson Ruck Entheogenic Research Institute and Archives, Taos, NM 87571, USA*
[6] *Department of Classical Studies, Boston University, Boston, MA 02215, USA*
[7] *Section of Ecology and Systematics, Department of Biology, National and Kapodistrian University of Athens (NKUA), Panepistimioupoli Zografou, 15784 Athens, Greece*

---

* Corresponding authors. Laboratory of Pharmacognosy and Chemistry of Natural Products, Department of Pharmacy, National and Kapodistrian University of Athens (NKUA), Panepistimioupoli Zografou, 15771 Athens, Greece. E-mail address: e.dadiotis@northeastern.edu (E. Dadiotis); magiatis@pharm.uoa.gr (P.M). Tel.: (+30) 210 727 4052

[+] These authors contributed equally to this work

Table of Contents

Supplementary Note 1: Ergot alkaloids – Structure, stereochemistry, classification and biosythesis. 4

Supplementary Note 2: Kykeon in ancient Greek texts – Variants, cultural context, standarization, technical and prepration aspects 5

Figure S1. ^{1}H NMR (400 MHz) spectrum of LSA (compound **1**) in $CDCl_3$ 6

Figure S2. Expanded region of the high-field resonances of rings C and D (*δ* 2.6–3.6 ppm) of LSA (compound **1**) from Figure S1 6

Figure S3. Expanded region of the low-field resonances (*δ* 6.5–7.3 ppm) of LSA (compound **1**) from Figure S1 7

Figure S4. ^{1}H NMR (400 MHz) spectrum of LSA (compound **1**) in DMSO-d_6 7

Figure S5. Expanded region of the high-field resonances of rings C and D (*δ* 2.4–3.5 ppm) of LSA (compound **1**) from Figure S4 8

Figure S6. Expanded region of the low-field resonances (*δ* 6.3–7.2 ppm) of LSA (compound **1**) from Figure S4 8

Figure S7. ^{13}C NMR (100 MHz) spectrum of LSA (compound **1**) in DMSO-d_6......... 9

Figure S8. ^{1}H–^{1}H COSY spectrum of LSA (compound **1**) in $CDCl_3$.......................... 9

Figure S9. ^{1}H–^{1}H COSY spectrum of LSA (compound **1**) in DMSO-d_6 10

Figure S10. ^{1}H–^{13}C HSQC spectrum of LSA (compound **1**) in DMSO-d_6................ 10

Figure S11. ^{1}H–^{13}C HMBC spectrum of LSA (compound **1**) in DMSO-d_6...............11

Figure S12. ^{1}H NMR (400 MHz) spectra of iso-LSA (compound **2**) in $CDCl_3$..........11

Figure S13. Expanded region of the high-field resonances of rings C and D (*δ* 2.5–3.6 ppm) of iso-LSA (compound **2**) from Figure S12 12

Figure S14. Expanded region of the low-field resonances (*δ* 6.5–7.3 ppm) of iso-LSA (compound **2**) from Figure S12 12

Figure S15. ^{1}H NMR (400 MHz) spectra of iso-LSA (compound **2**) in DMSO-d_6 ... 13

Figure S16. Expanded region of the high-field resonances of rings C and D (*δ* 2.4–3.5 ppm) of iso-LSA (compound **2**) from Figure S15 13

Table of Contents (cont.)

Figure S17. Expanded region of the low-field resonances (δ 6.4–7.3 ppm) of iso-LSA (compound **2**) from Figure S15 14

Figure S18. ^{13}C NMR (100 MHz) spectrum of iso-LSA (compound **2**) in DMSO-d_6 14

Figure S19. ^{1}H–^{1}H COSY spectrum of iso-LSA (compound **2**) in $CDCl_3$ 15

Figure S20. ^{1}H–^{1}H COSY spectrum of iso-LSA (compound **2**) in DMSO-d_6 15

Figure S21. ^{1}H–^{13}C HSQC spectrum of iso-LSA (compound **2**) in DMSO-d_6 16

Figure S22. ^{1}H–^{13}C HMBC spectrum of iso-LSA (compound **2**) in DMSO-d_6 16

Figure S23. a) Conidia at 400× (DIC; scale bar = 10 μm), b) sclerotia (scale in cm), and c) inoculated sclerotia on PDA in a 9 cm Petri plate after 1 week. All images from strain ATHUM 10382. 17

Table S1. Sequence of the ITS rDNA region of ATHUM 10382 15

Figure S24. Total ion chromatogram (TIC) and extracted ion chromatograms (EICs) of reference standards 18

Table S2. UHPLC/Q-TOF-HRMS quantitation results for LSA and iso-LSA in the extracts and related calculations 19

Figure S25. Proposed mechanism of the chemical transformation of the ergopeptines ergokryptine and ergocristine into LSA/iso-LSA and non-toxic secondary by-products 20

Table S3. Results of effect tests and parameter estimates, together with their levels of significance, derived from the model equations employed for LSA 21

Table S4. Results of effect tests and parameter estimates, together with their levels of significance, derived from the model equations employed for iso-LSA 21

Table S5. Summary of fit based on the model equations applied for LSA and iso-LSA. 21

References 22

Supplementary Note 1: Ergot alkaloids – Structure, stereochemistry, classification and biosythesis.

General Description

Over 80 ergot alkaloids (EAs) have been isolated from diverse natural sources, primarily from members of the genus *Claviceps* infecting higher plants, but also from other fungal taxa occurring as symbionts or endophytes of their hosts[1–4]. EAs are a structurally diverse class of alkaloids biosynthetically derived from tryptophan (Trp) and dimethylallyl pyrophosphate (DMAPP)[5], along with their semi-synthetic derivatives and synthetic analogues. All share the ergoline ring system as their core structure[6].

Stereochemistry

- Most EAs are methylated at N-6 and substituted at C-8[7,8]
- They commonly feature a double bond at either C8–C9 ($\Delta^{8,9}$-ergolenes) or C9–C10 ($\Delta^{9,10}$-ergolenes)[6].
- Stereocenters occur at C-5 and C-8 (or C-10), allowing up to four stereoisomers per E[6].
- In naturally occurring EAs, the C-5 stereocenter is invariably (*R*) configuration[8].
- At C-8, (*R*)-epimers (i.e., (+)-*d*-lysergic acid derivatives) are designated with the suffix "-ine" and they typically exhibit high pharmacological activity at adrenergic, dopaminergic, and serotonergic receptors[9–11].
- The usually inactive (*S*)-epimers carry the suffix "-inine" or the prefix "iso-"[10].
- C-8 epimers are interconvertible and prone to epimerization under heat, light, or extreme pH[7].
- Historical terms like *α* and *β* epimers refer to the same C-8 stereochemistry but are inconsistent with modern nomenclature[11].

Structural Classes of EAs[4,7,8]

EAs are categorized into four main biosynthetically related groups:

1. Clavines
   - Ergoline structure or tricyclic precursors of ergoline with an open D ring.
   - Do not yield lysergic acid upon hydrolysis.
   - Subdivided into tetracyclic, tricyclic, or rearranged subclasses.

2. Lysergamides (Ergoamides)
   - Simple lysergic acid derivatives (primary or secondary amides).
   - Include compounds such as LSA and LSD analogues.

3. Ergopeptines
   - Lysergic acid linked to three amino acids forming a tricyclic peptide.
   - Classified into subgroups based on peptide composition.
   - Often referred to as cyclol ergot alkaloids (CEA).

4. Ergopeptams
   - Structurally similar to ergopeptines but with a bicyclic peptide.
   - Classified into subgroups based on peptide composition.
   - Often annotated as lactam ergot alkaloids (LEA).

(Ergopeptines and ergopeptams are collectively called peptidic EAs or ergopeptides)

Supplementary Note 2: Kykeon in ancient Greek texts – Variants, cultural context, standarization, technical and prepration aspects.

There are many "versions" or variants of the kykeon, described in ancient Greek manuscripts beyond the Eleusinian formulation. Despite their variations, all share a common nucleus: mainly prepared by women with the inclusion of barley, barley groats or barley flour referred to in ancient Greek as *ἄλφι, ἄλφιτον, or ἄλφιτα*[12], with the resulting mixture acting as a drug, referred to in ancient Greek as *φάρμακον* (from *φέρω* "to bring" and *ἄκος* "cure/remedy"), highlighting kykeon's dual role as both a therapeutic agent and a potentially intoxicating and ritualistic substance[13]. Such examples in ancient Greek literature, beyond the Eleusinian formulation, are the following:

1. A tonic mixed restorative potion prepared by Hecamede and given to Nestor and the wounded Machaon, when they left the fray of battle of Troy, referred to in book 11 line 624 (or 638–641 depending on edition) in Homer's "The Illiad". The drink includes barley-meal, goat-cheese grated into a wine (Pramnian wine) base.
2. The magic intoxicating mixed potion crafted by the demi-goddess and witch Kirke (Circe) and used to drug Odysseus and transform his companions into swine as referred to in book 10 lines 234–237, 290 and 316-317 of Homer's "The Odyssey". Kirke also adds some honey and pours her magic potion into it.
3. The kykeon referred by the ancient Greek poet Hipponax as a drug or remedy for his soul torments[13].
4. Theophrastus (319 BC) refers to the drink in his "Characters" (IV, 2-3), mentioning a drunk farmer whose thyme-scented breath annoyed the Assembly in the Ecclesia.
5. Its digestive properties are mentioned in Aristophanes' "Peace" (V. 712) (421 BC), when Hermes suggests it to the hero who had eaten too many dried fruits and nuts.

<u>Scale and standardization:</u> The preparation of kykeon for thousands of initiates would have been facilitated by large-capacity vessels, called *λέβητες* (*lebetes*; cauldrons). Large-scale preparation naturally acts as an empirical "chemical buffer" averaging out potential variations and ensuring a more consistent final product across the entire batch. In an era before analytical chemistry, such consistency was likely maintained through hereditary ritual protocols and the use of fixed ingredient ratios (e.g., specific weights of ash per volume of water), a form of empirical standardization typical of ancient traditional craftsmanship.

<u>Other technical and preparation aspects:</u> Beyond the ingredients, the preparation of barley-based beverages in antiquity, such as *πτισάνη* (*ptisane*; barley water) and various medicinal decoctions, frequently involved heating or boiling to ensure the softening of the grain and the extraction of nutrients. Hippocrates (On Regimen in Acute Diseases) provides detailed instructions for boiling barley to create a smooth, homogenized mixture. In the context of the kykeon, while the Homeric Hymn does not explicitly detail the temperature, the term *κῠκάω* (*kykao*; to stir/mix) implies a process of homogenization which, in ancient pharmacology, was typically facilitated by heat to ensure that the solid components (barley meal) and additives (herbs, honey cheese and/or wine, as stated above) formed a uniform suspension.

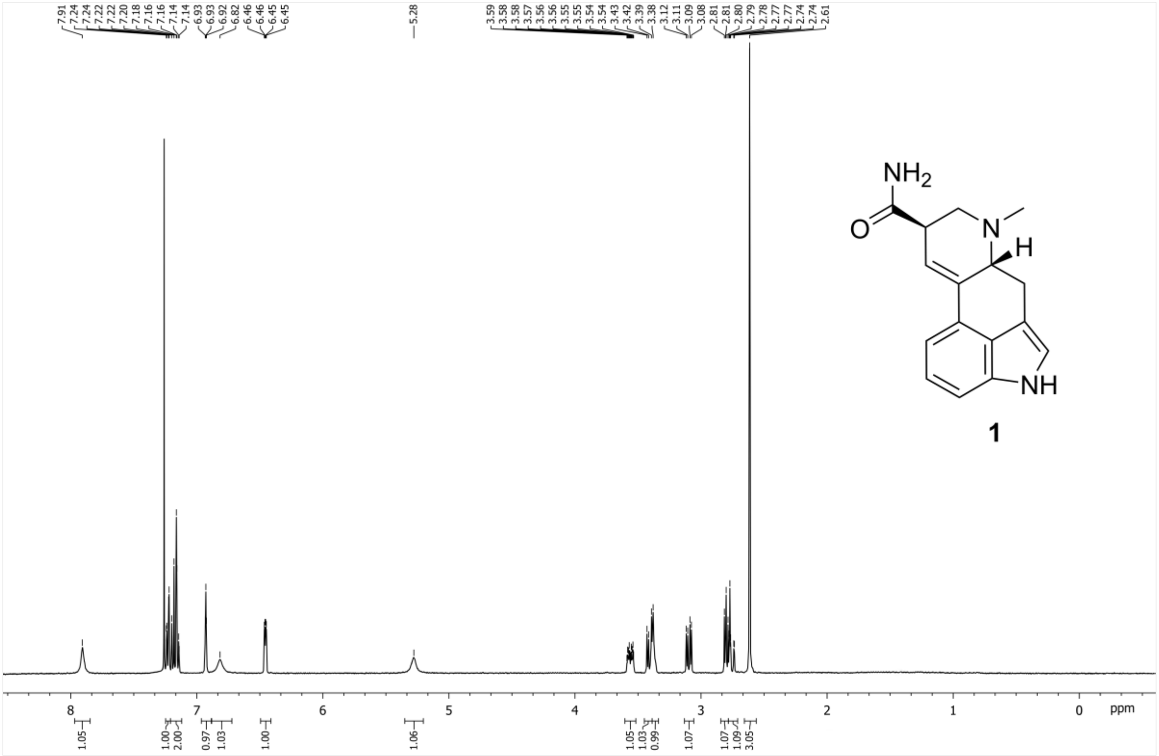

Figure S1. ^{1}H NMR (400 MHz) spectrum of LSA (compound **1**) in $CDCl_3$

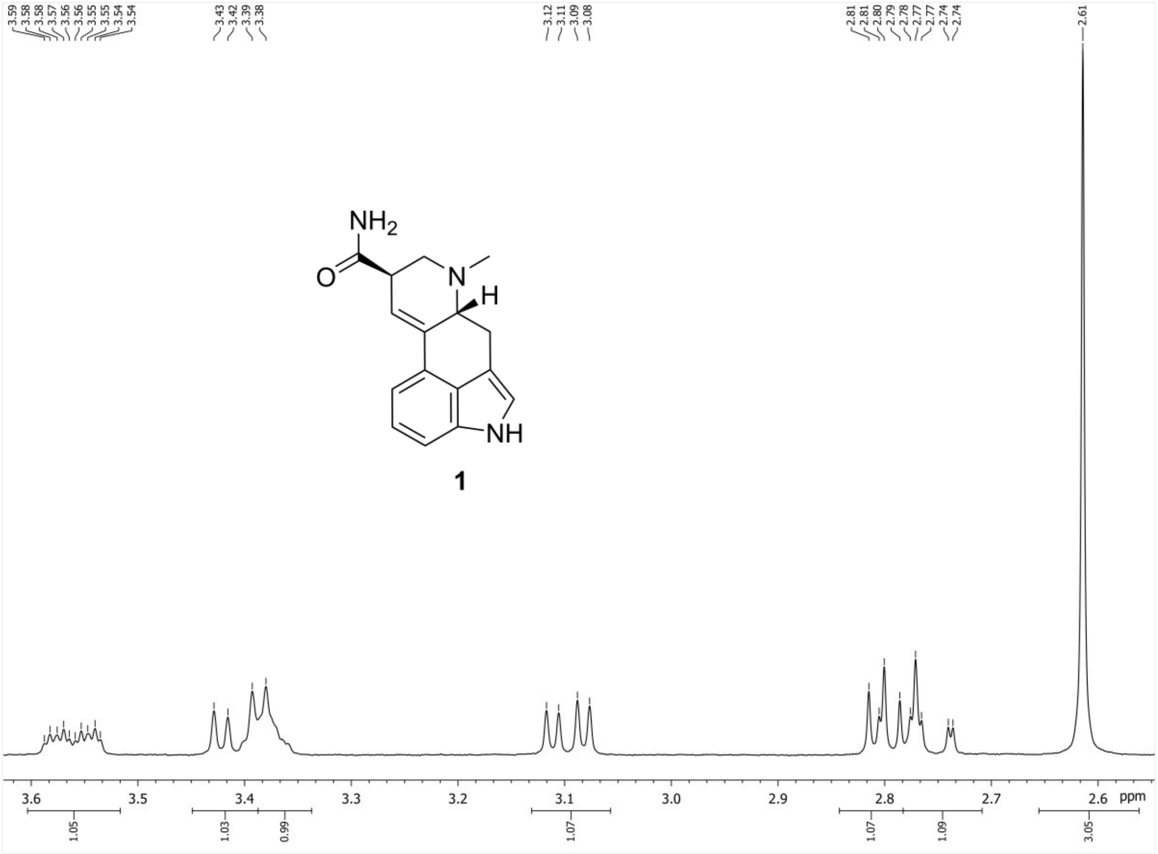

Figure S2. Expanded region of the high-field resonances of rings C and D (*δ* 2.6–3.6 ppm) of LSA (compound **1**) from Figure S1

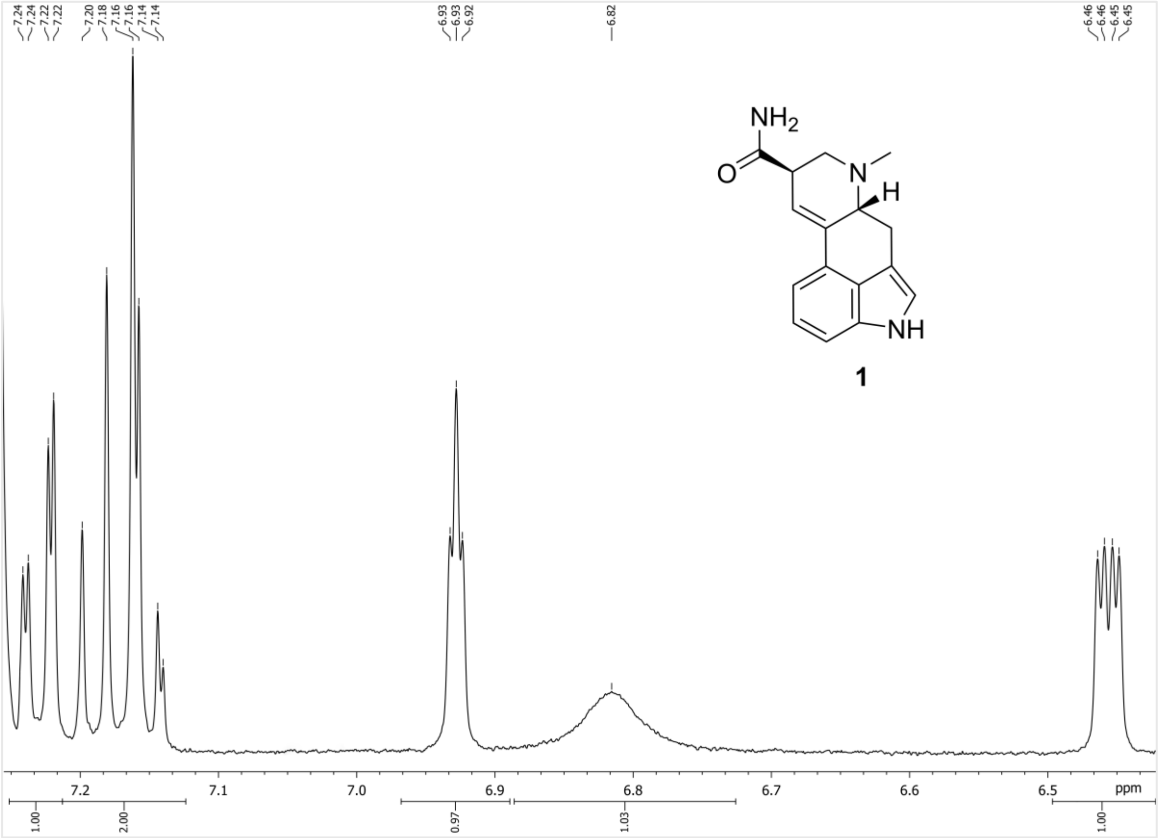

Figure S3. Expanded region of the low-field resonances (*δ* 6.5–7.3 ppm) of LSA (compound **1**) from Figure S1

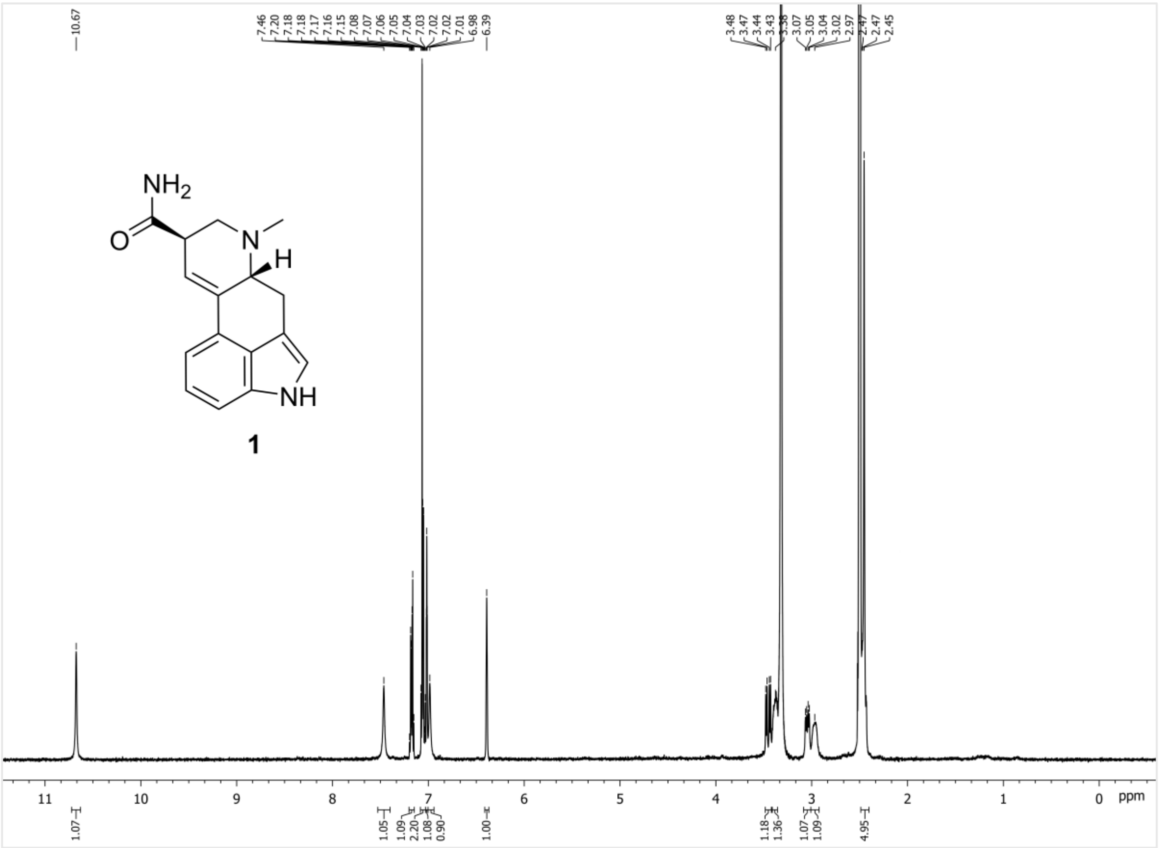

Figure S4. ^{1}H NMR (400 MHz) spectrum of LSA (compound **1**) in DMSO-d_6

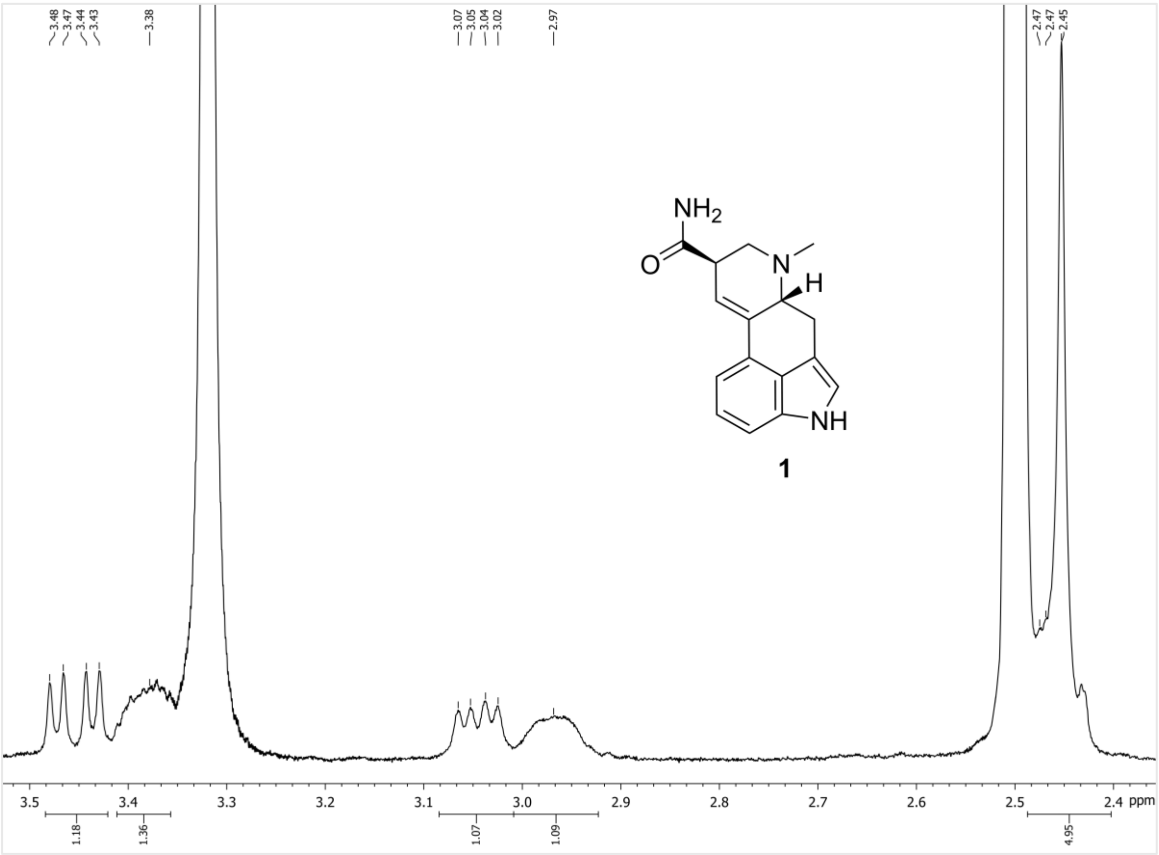

Figure S5. Expanded region of the high-field resonances of rings C and D (δ 2.4–3.5 ppm) of LSA (compound **1**) from Figure S4

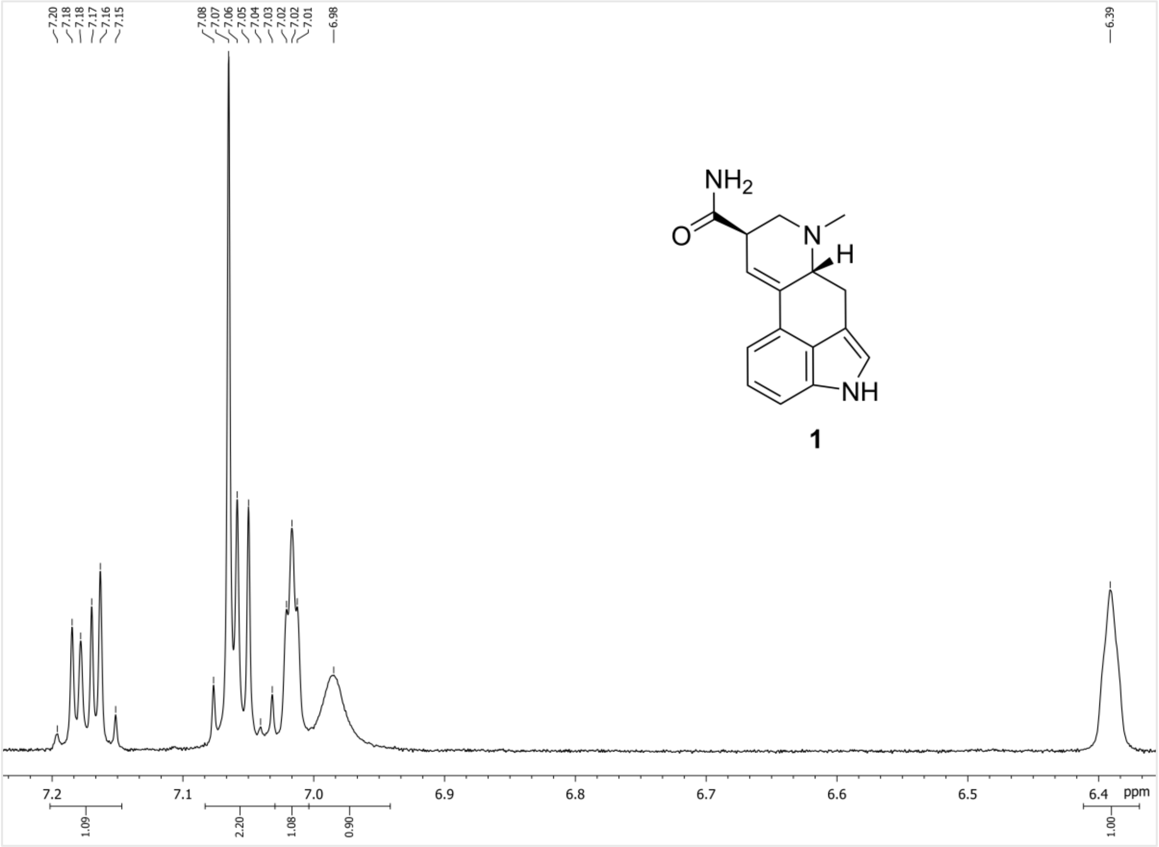

Figure S6. Expanded region of the low-field resonances (δ 6.3–7.2 ppm) of LSA (compound **1**) from Figure S4

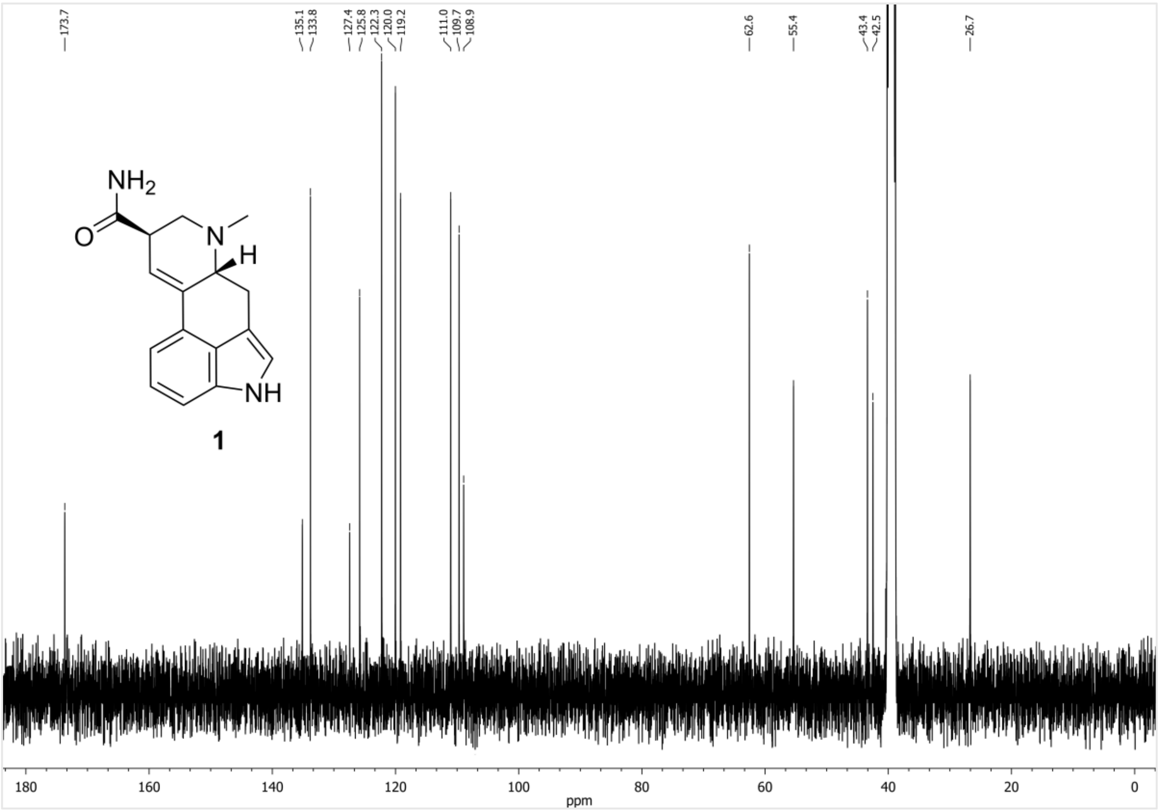

Figure S7. ^{13}C NMR (100 MHz) spectrum of LSA (compound **1**) in DMSO-d$_6$

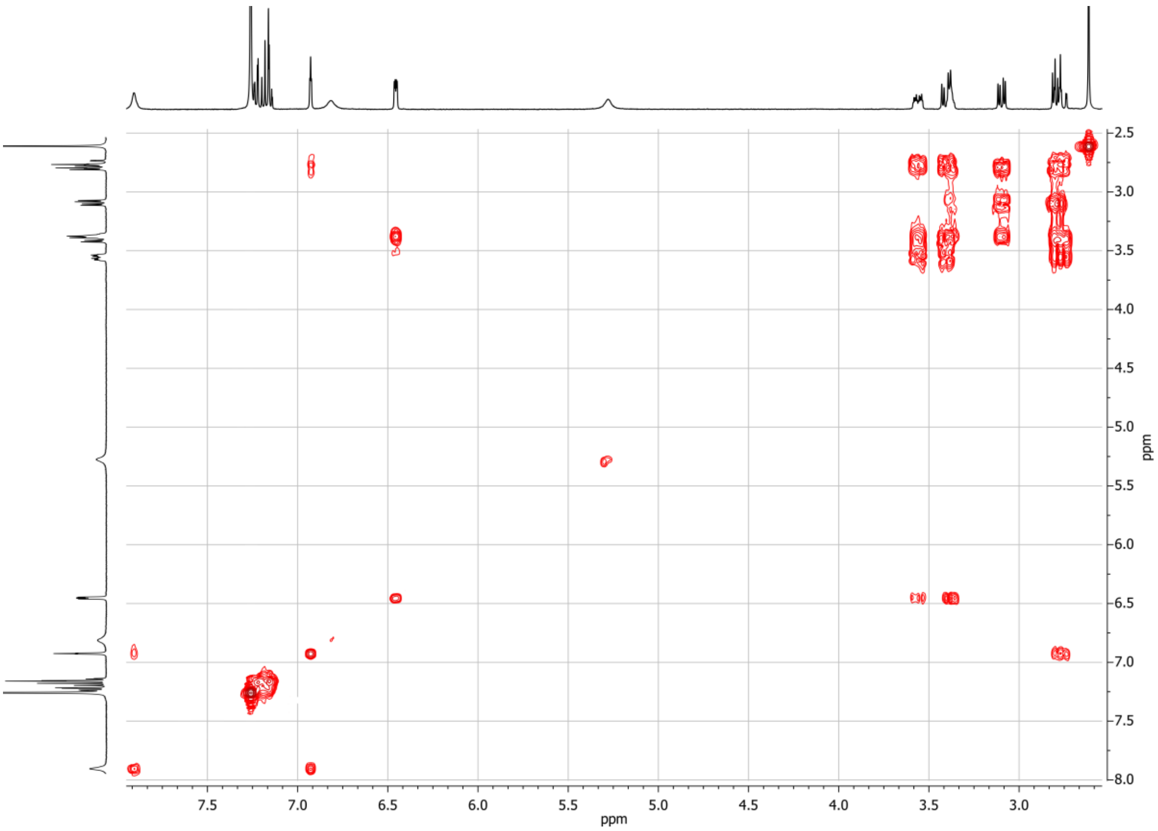

Figure S8. ^{1}H–^{1}H COSY spectrum of LSA (compound **1**) in $CDCl_3$

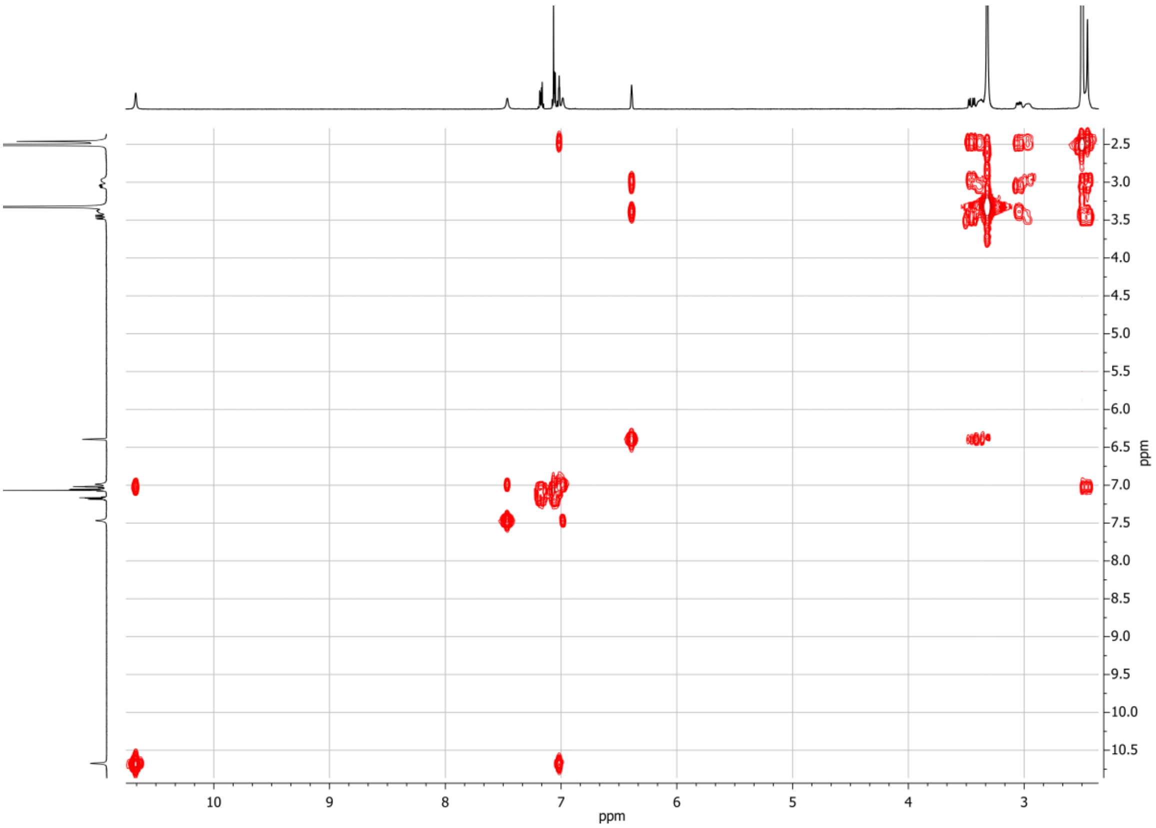

Figure S9. ^{1}H–^{1}H COSY spectrum of LSA (compound **1**) in DMSO-d_6

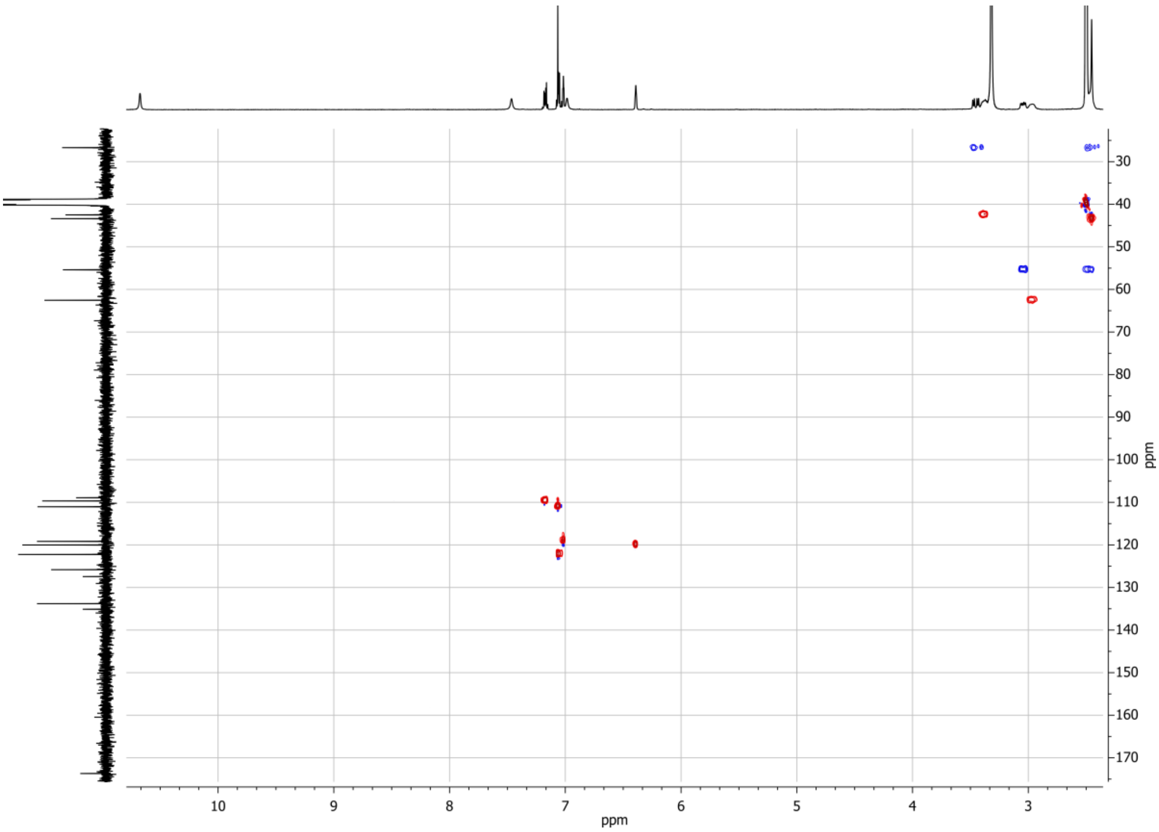

Figure S10. ^{1}H–^{13}C HSQC spectrum of LSA (compound **1**) in DMSO-d_6

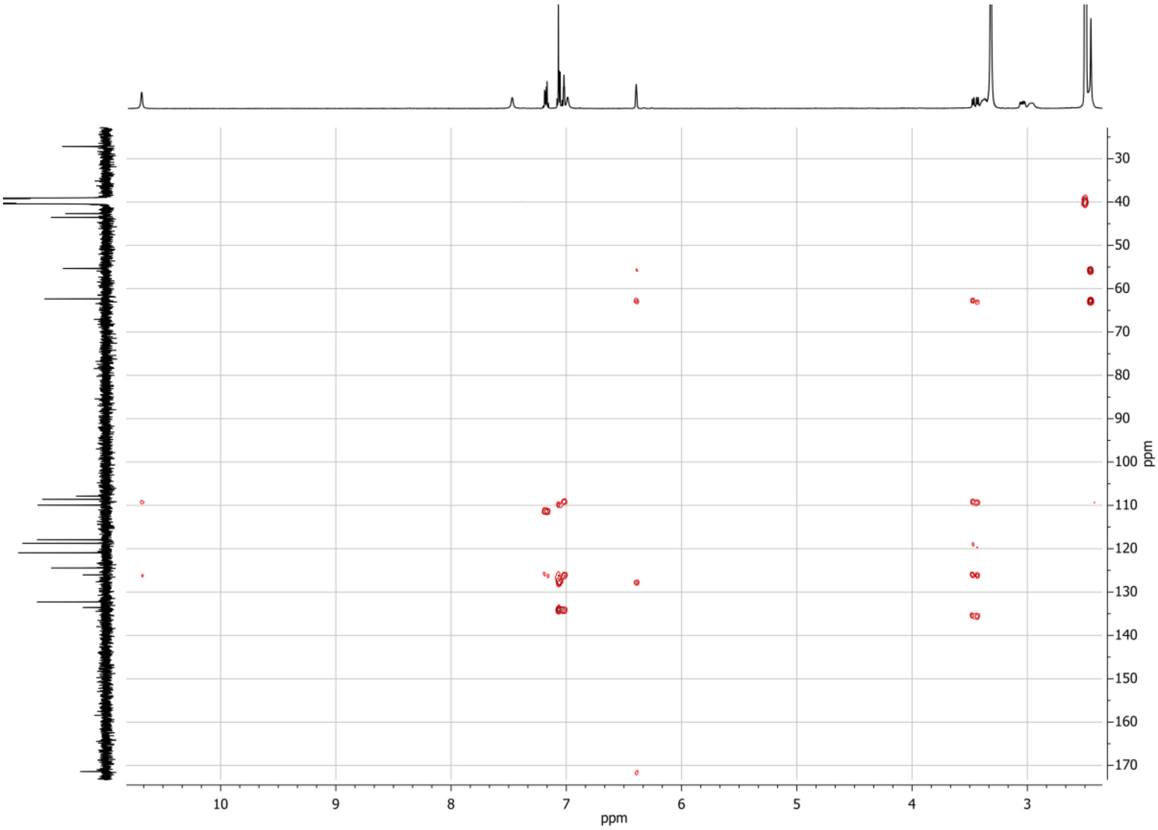

Figure S11. ^{1}H–^{13}C HMBC spectrum of LSA (compound **1**) in DMSO-d_6

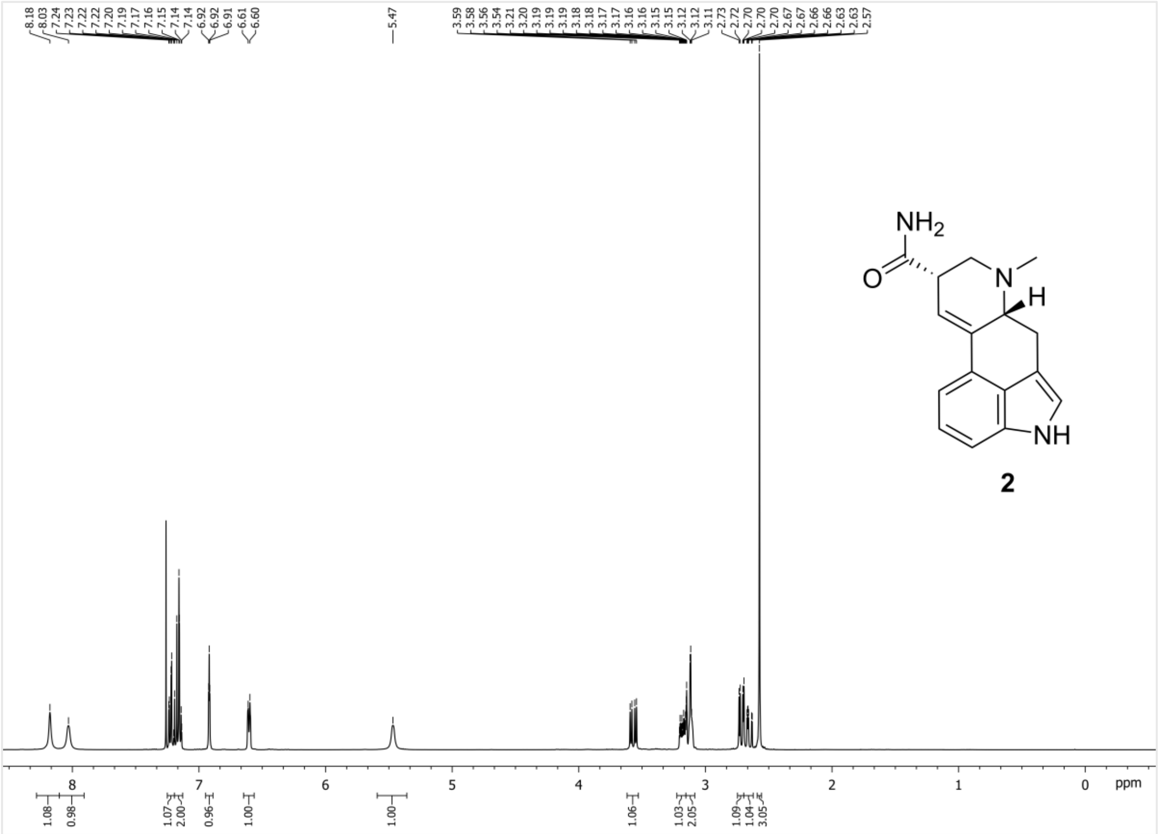

Figure S12. ^{1}H NMR (400 MHz) spectra of iso-LSA (compound **2**) in $CDCl_3$

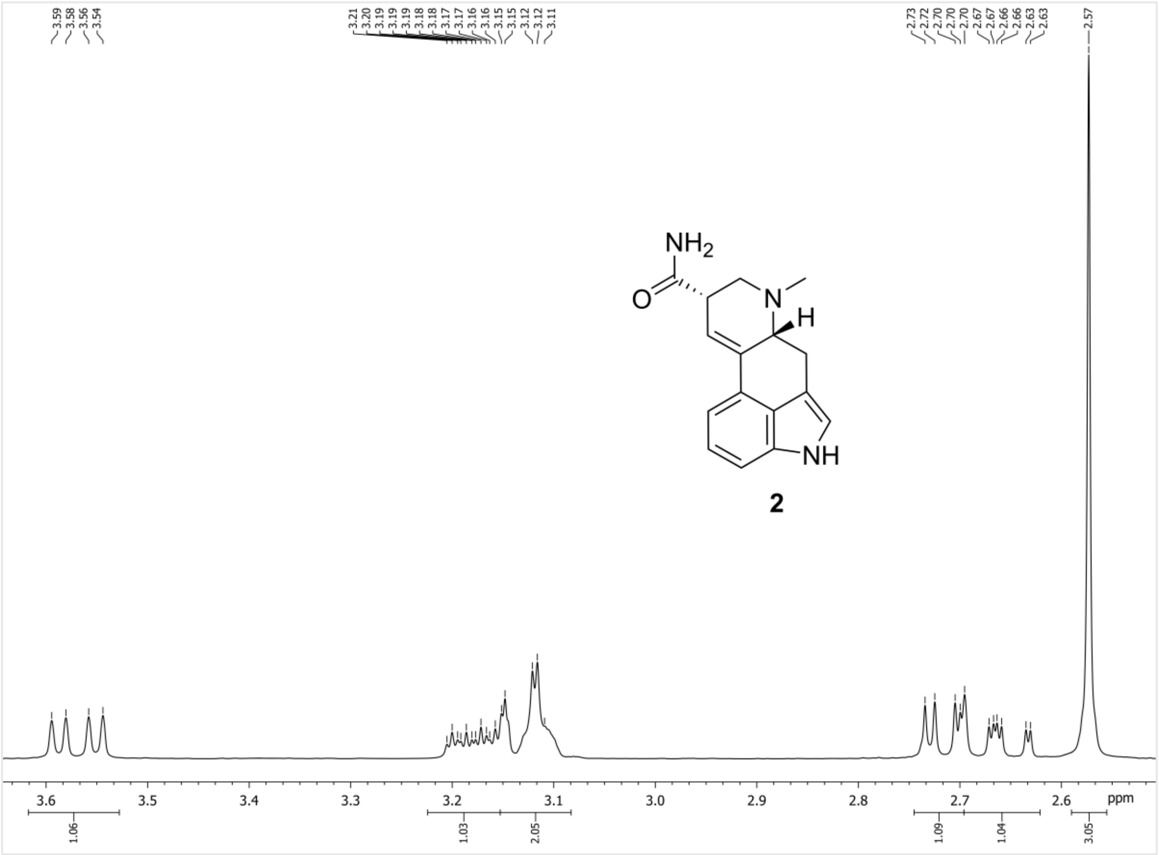

Figure S13. Expanded region of the high-field resonances of rings C and D (*δ* 2.5–3.6 ppm) of iso-LSA (compound **2**) from Figure S12

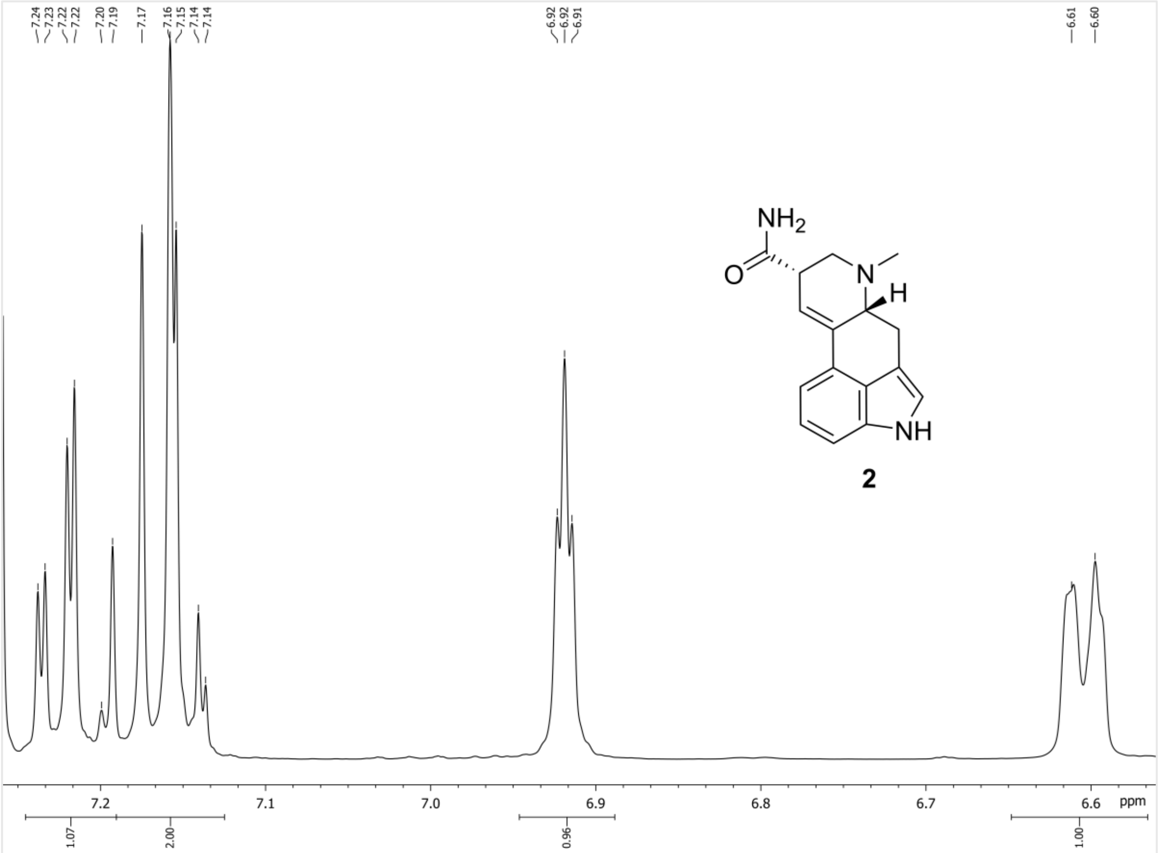

Figure S14. Expanded region of the low-field resonances (*δ* 6.5–7.3 ppm) of iso-LSA (compound **2**) from Figure S12

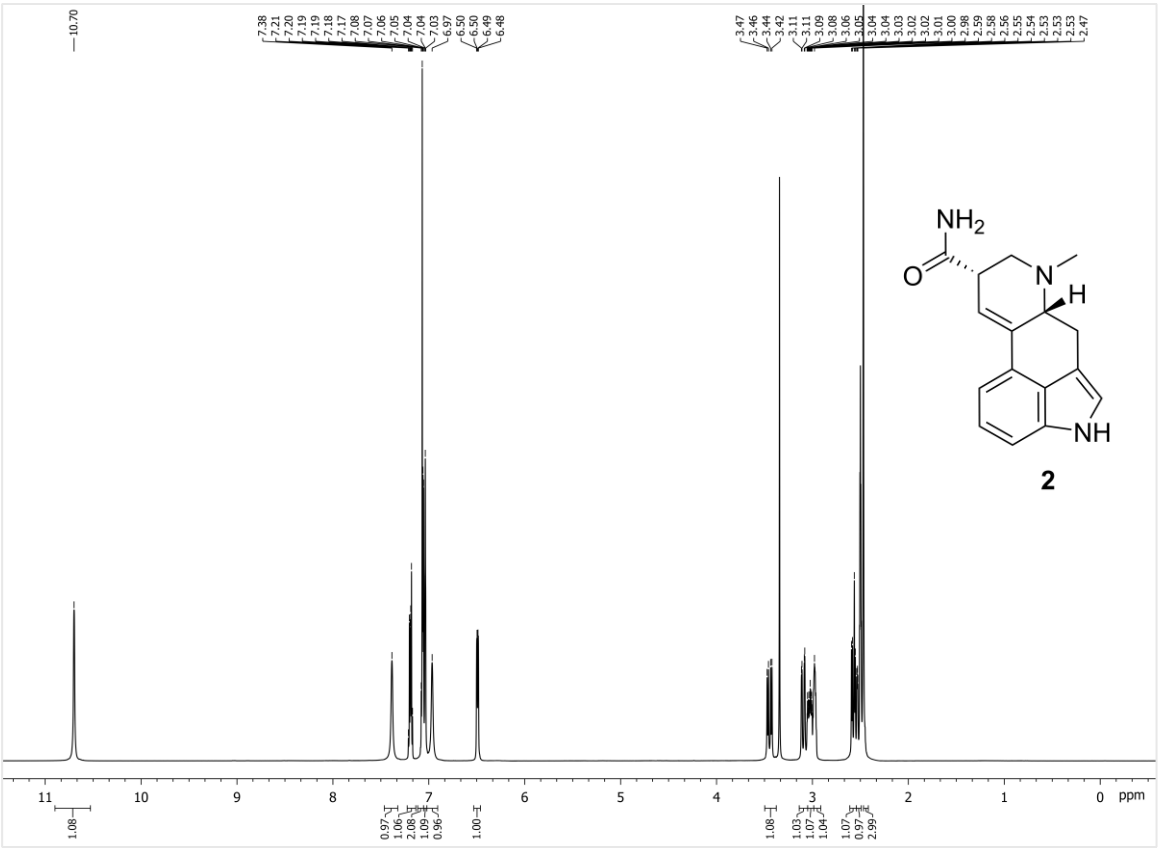

Figure S15. ^{1}H NMR (400 MHz) spectra of iso-LSA (compound **2**) in DMSO-d_6

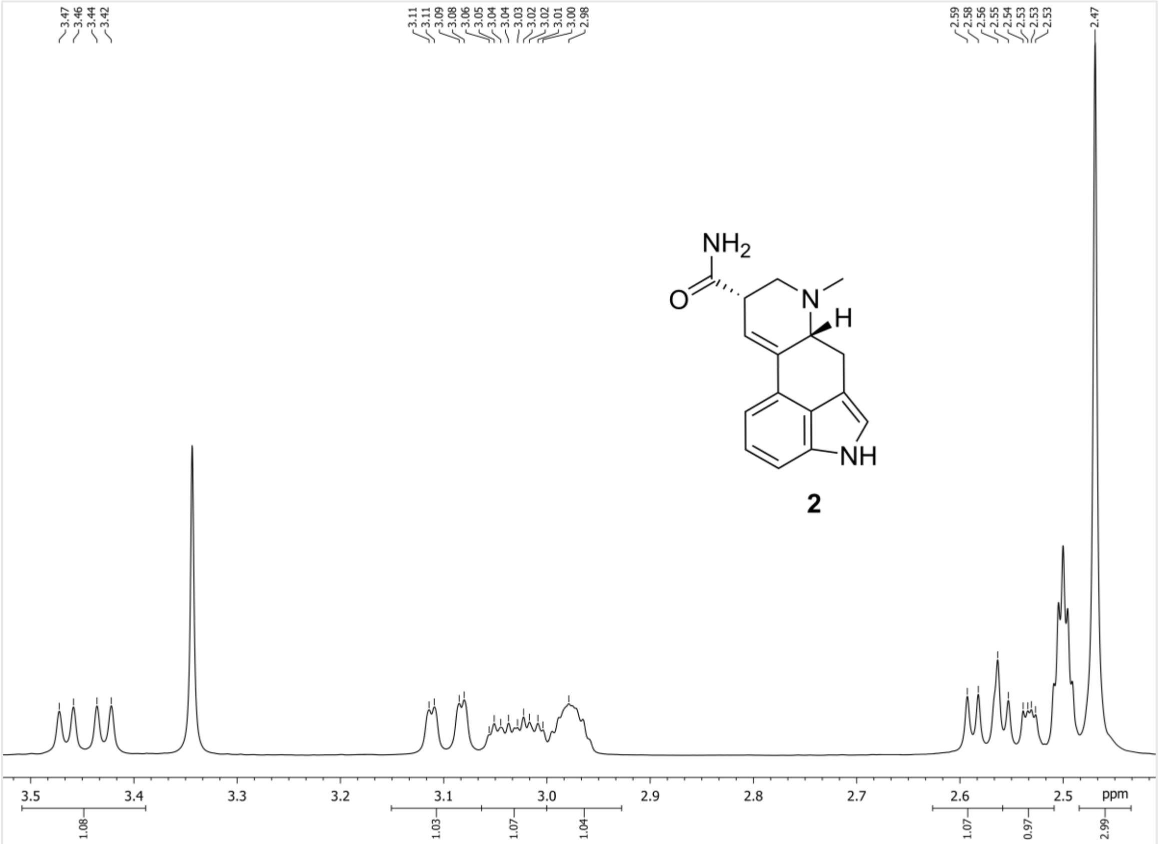

Figure S16. Expanded region of the high-field resonances of rings C and D (δ 2.4–3.5 ppm) of iso-LSA (compound **2**) from Figure S15

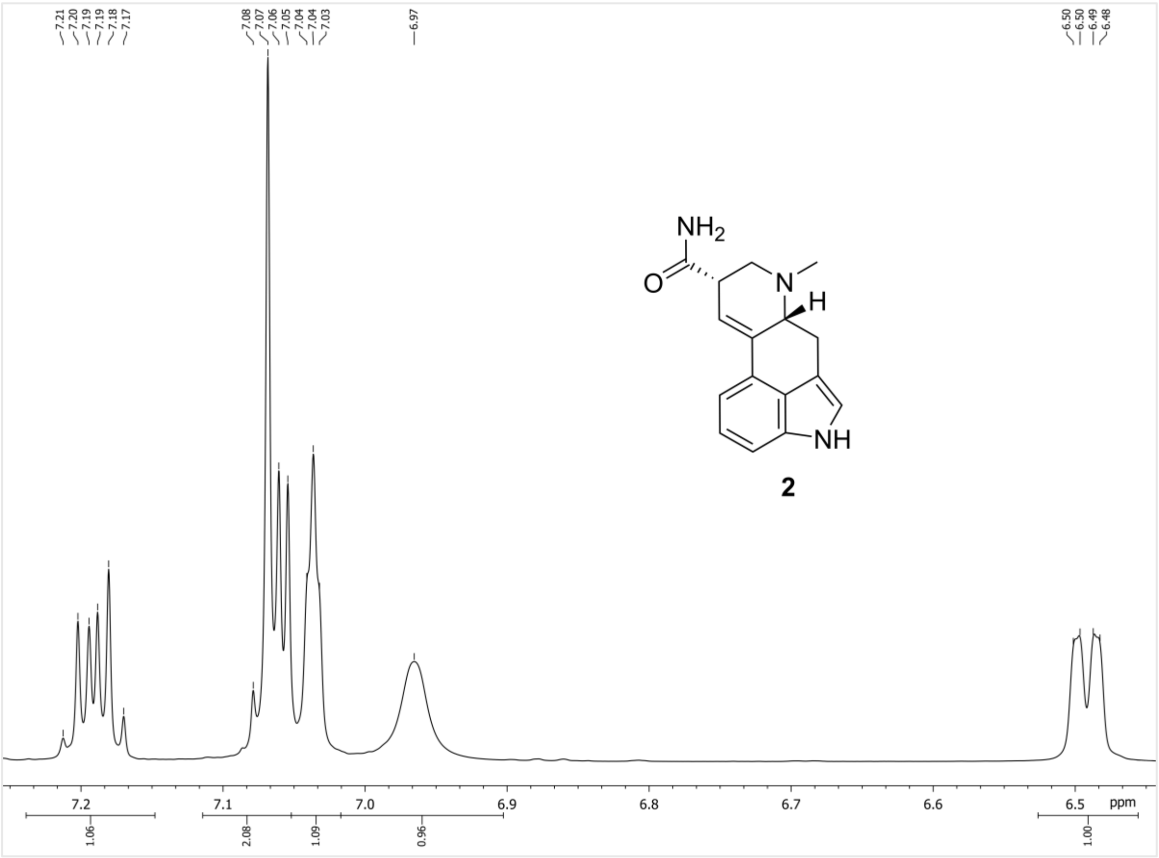

Figure S17. Expanded region of the low-field resonances (δ 6.4–7.3 ppm) of iso-LSA (compound **2**) from Figure S15

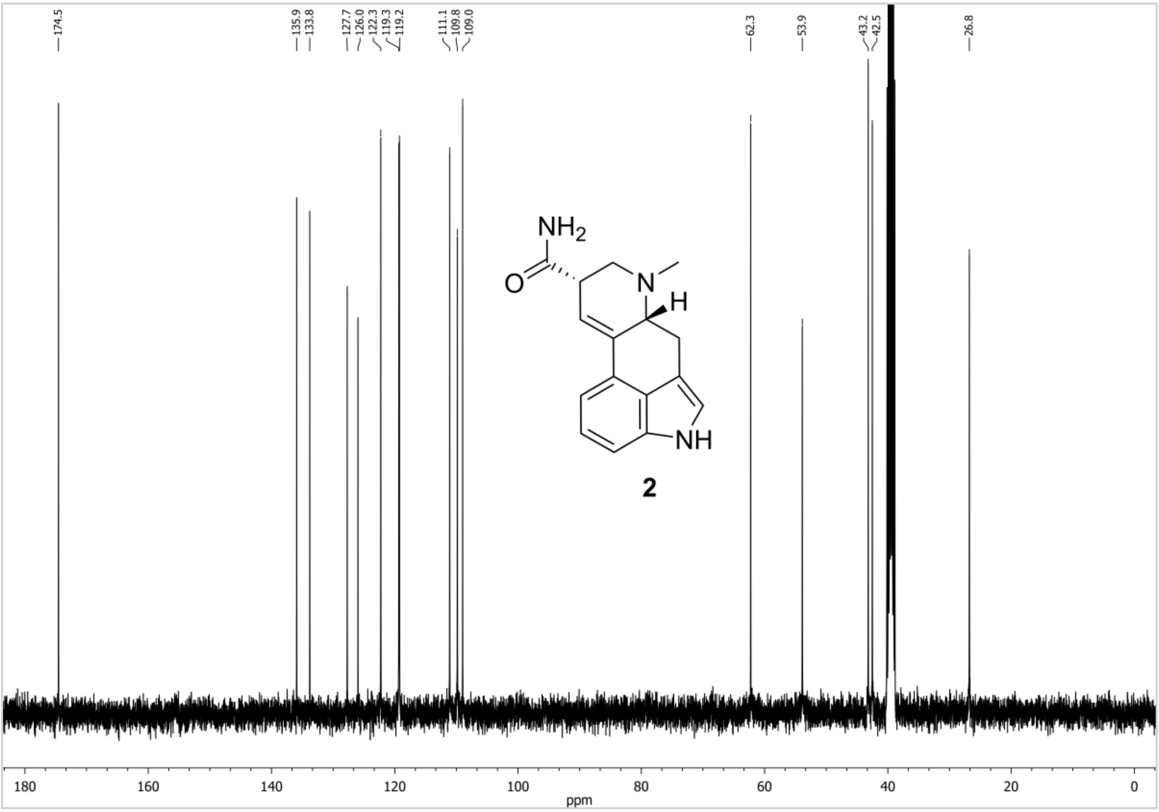

Figure S18. ^{13}C NMR (100 MHz) spectrum of iso-LSA (compound **2**) in DMSO-d_6

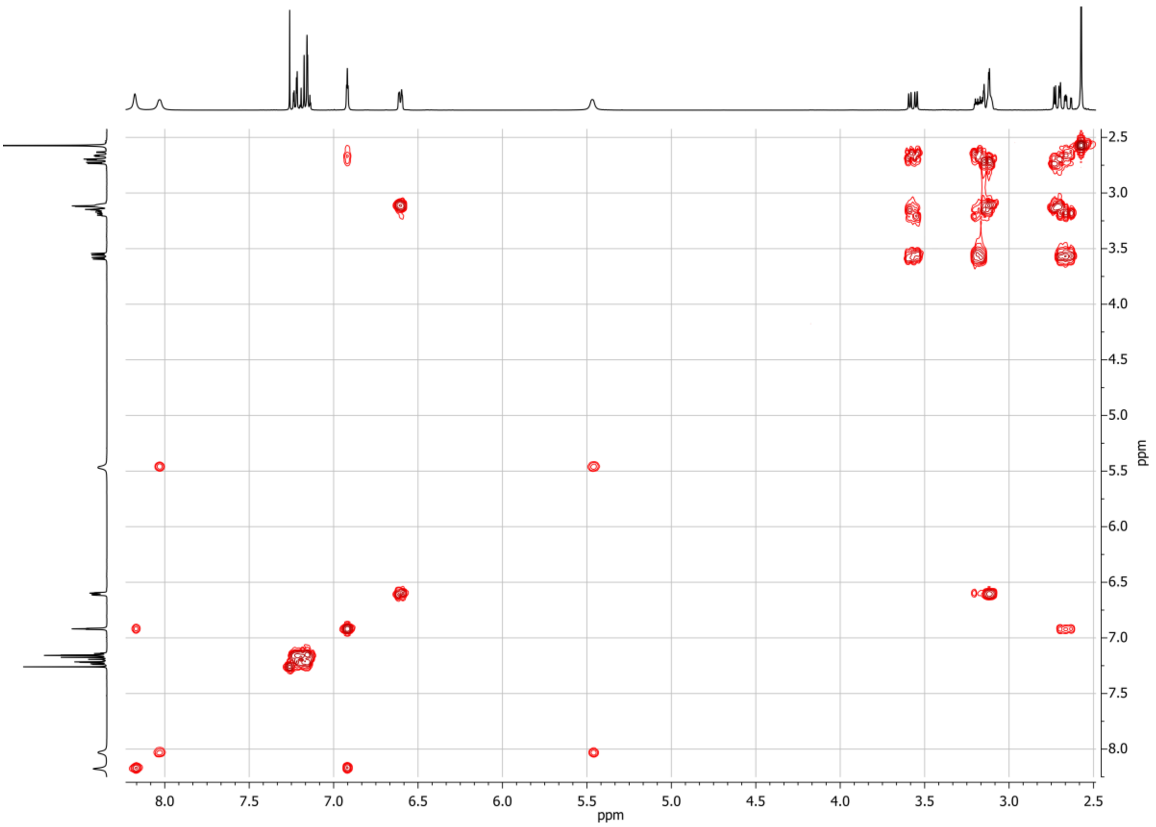

Figure S19. ^{1}H–^{1}H COSY spectrum of iso-LSA (compound **2**) in $CDCl_3$

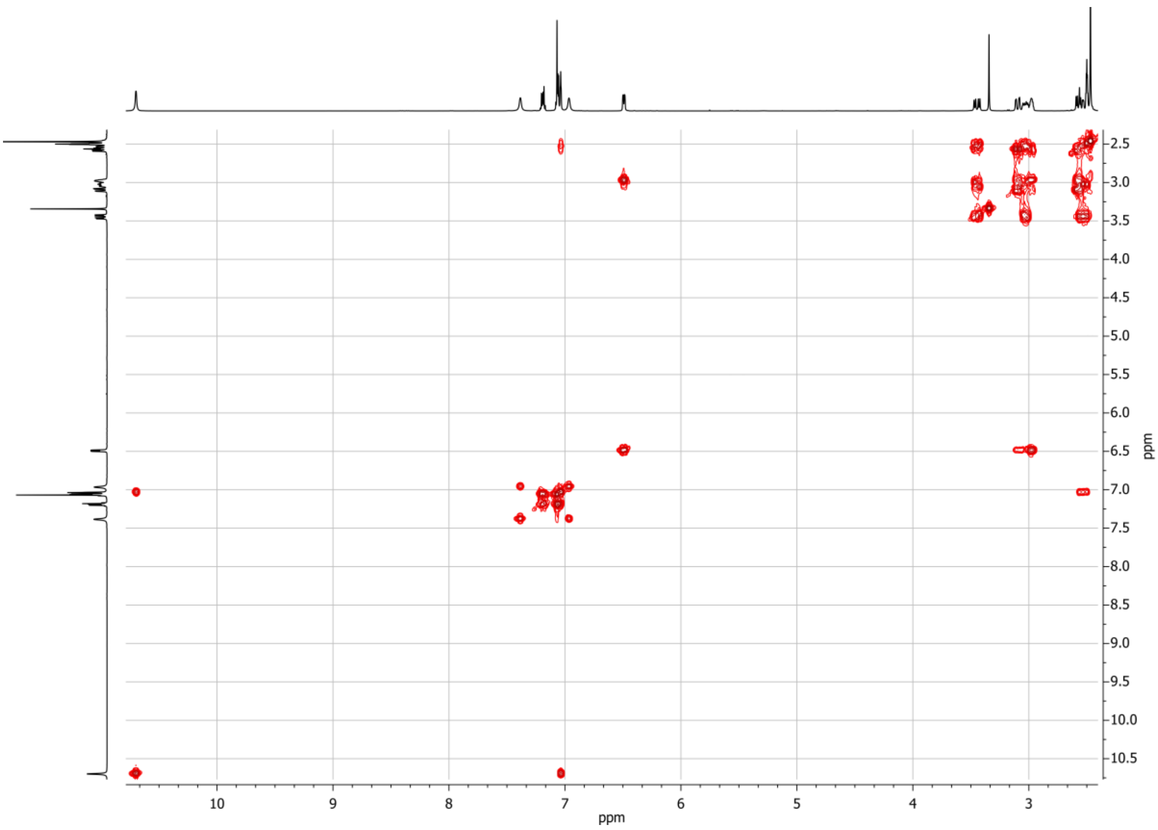

Figure S20. ^{1}H–^{1}H COSY spectrum of iso-LSA (compound **2**) in DMSO-d_6

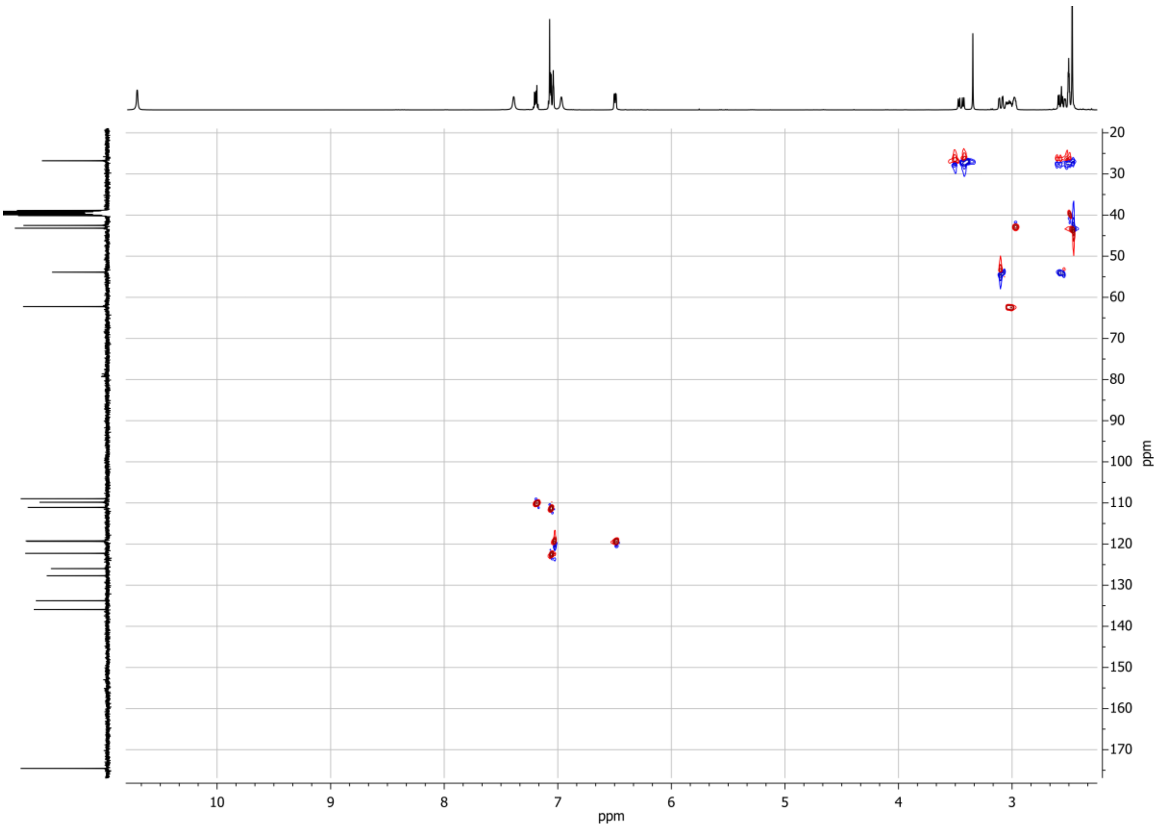

Figure S21. ^{1}H–^{13}C HSQC spectrum of iso-LSA (compound **2**) in DMSO-d_6

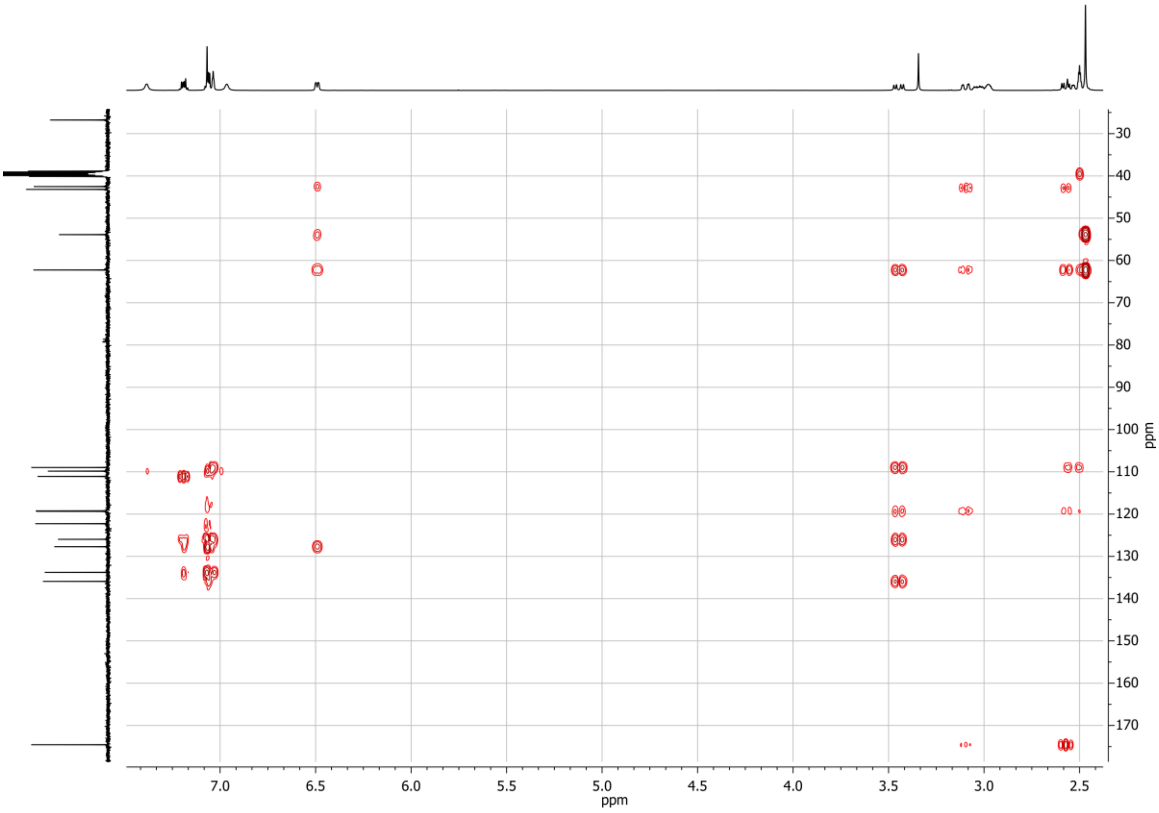

Figure S22. ^{1}H–^{13}C HMBC spectrum of iso-LSA (compound **2**) in DMSO-d_6

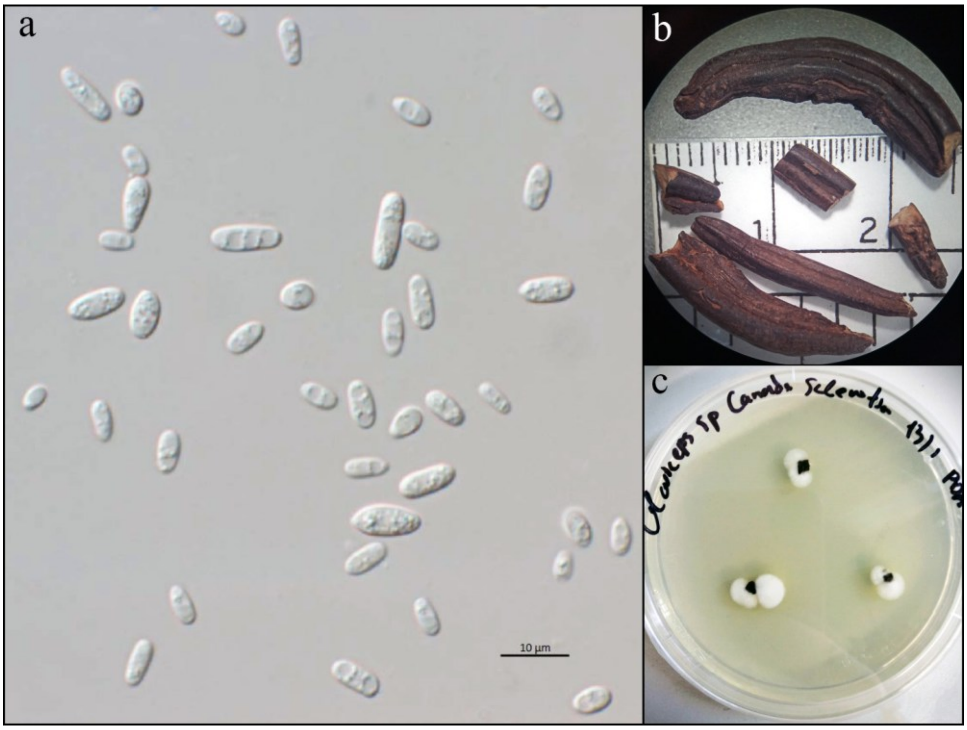

Figure S23. a) Conidia at 400× (DIC; scale bar = 10 μm), b) sclerotia (scale in cm), and c) inoculated sclerotia on PDA in a 9 cm Petri plate after 1 week. All images from strain ATHUM 10382.

Table S1. Sequence of the ITS rDNA region of ATHUM 10382 (GenBank: PX700742.1)

	rDNA-ITS region (5′→3′; contig length = 738 bases)					
1	GCCCGTCGCT	ACTACCGATT	GAATGGCTCA	GTGAGGCGTC	CGGACTGGCC	CAGAGAGGTG
61	GGCAACTACC	ACTCAGGGCC	GGAAAGCTCT	CCAAACTCGG	TCATTTAGAG	GAAGTAAAAG
121	TCGTAACAAG	GTCTCCGTTG	GTGAACCAGC	GGAGGGATCA	TTACCGAGTT	TACAACTCCC
181	AAACCCACTG	TGAACTTATA	CCCAAAACGT	TGCCTCGGCG	GGCACAGCGG	TACCCGAGCC
241	CCCCGCAAGG	GAGGCAGAGG	CGCCCGCCCG	CCAGGGGACC	AAAACTCTTC	TGTATACCCA
301	TAGCGGCATG	TCTGAGTGGA	TTTACAAACA	AATGAATCAA	AACTTTCAAC	AACGGATCTC
361	TTGGTTCTGG	CATCGATGAA	GAACGCAGCG	AAATGCGATA	CGTAATGTGA	ATTGCAGAAT
421	TCAGTGAATC	ATCGAATCTT	TGAACGCACA	TTGCGCCCGC	CAGTATTCTG	GCGGGCATGC
481	CTGTTCGAGC	GTCATTTCAA	CCCTCAAGCC	CTGCTTGGTG	TTGGGGACCG	GCTCAGCGGG
541	TGCGGGCTTC	GGCCCGCCCC	GTGCCGCCCC	CGAAATGGAT	CGGCGGTCTC	GTCGCAGCCT
601	TCTTTGCGTA	GTAACATACC	ACCTCGCAAC	AGGAGCGCGG	CGCGGCCACT	GCCGTAAAAC
661	GCCCAACTTT	TTAGAGTTGA	CCTCGAATCA	GGTAGGAATA	CCCGCTGAAC	TTAAGCATAT
721	CAATAAGCGG	AGGAAGAC				

TIC of reference standards mixed solution, 0.1 μg mL^{-1} each in acetonitrile. Chromatographic conditions as described in Section 2.8.

EIC for $[M+H]^+$ *m/z* 269.1444
Molecular formula: $C_{16}H_{17}N_3O$
peak a: isoergine (iso-LSA)
peak b: ergine (LSA)

EIC for $[M+H]^+$ *m/z* 326.1868
Molecular formula: $C_{19}H_{23}N_3O_2$
peak a: ergometrine (Em)
peak b: ergometrinine (Emn)

EIC for $[M+H]^+$ *m/z* 548.2872
Molecular formula: $C_{30}H_{37}N_5O_5$
peak a: ergosinine (Esn)
peak b: ergosine (Es)

EIC for $[M+H]^+$ *m/z* 562.3029
Molecular formula: $C_{31}H_{39}N_5O_5$
peak a: ergocornine (Eco)
peak b: ergocorninine (Econ)

EIC for $[M+H]^+$ *m/z* 576.3185
Molecular formula: $C_{32}H_{41}N_5O_5$
peak a: ergokryptine (Ekr)
peak b: ergokryptinine (Ekrn)

EIC for $[M+H]^+$ *m/z* 582.2716
Molecular formula: $C_{33}H_{35}N_5O_5$
peak a: ergotaminine (Etn)
peak b: ergotamine (Et)

EIC for $[M+H]^+$ *m/z* 610.3029
Molecular formula: $C_{35}H_{39}N_5O_5$
peak a: ergocristine (Ecr)
peak b: ergocristinine (Ecrn)

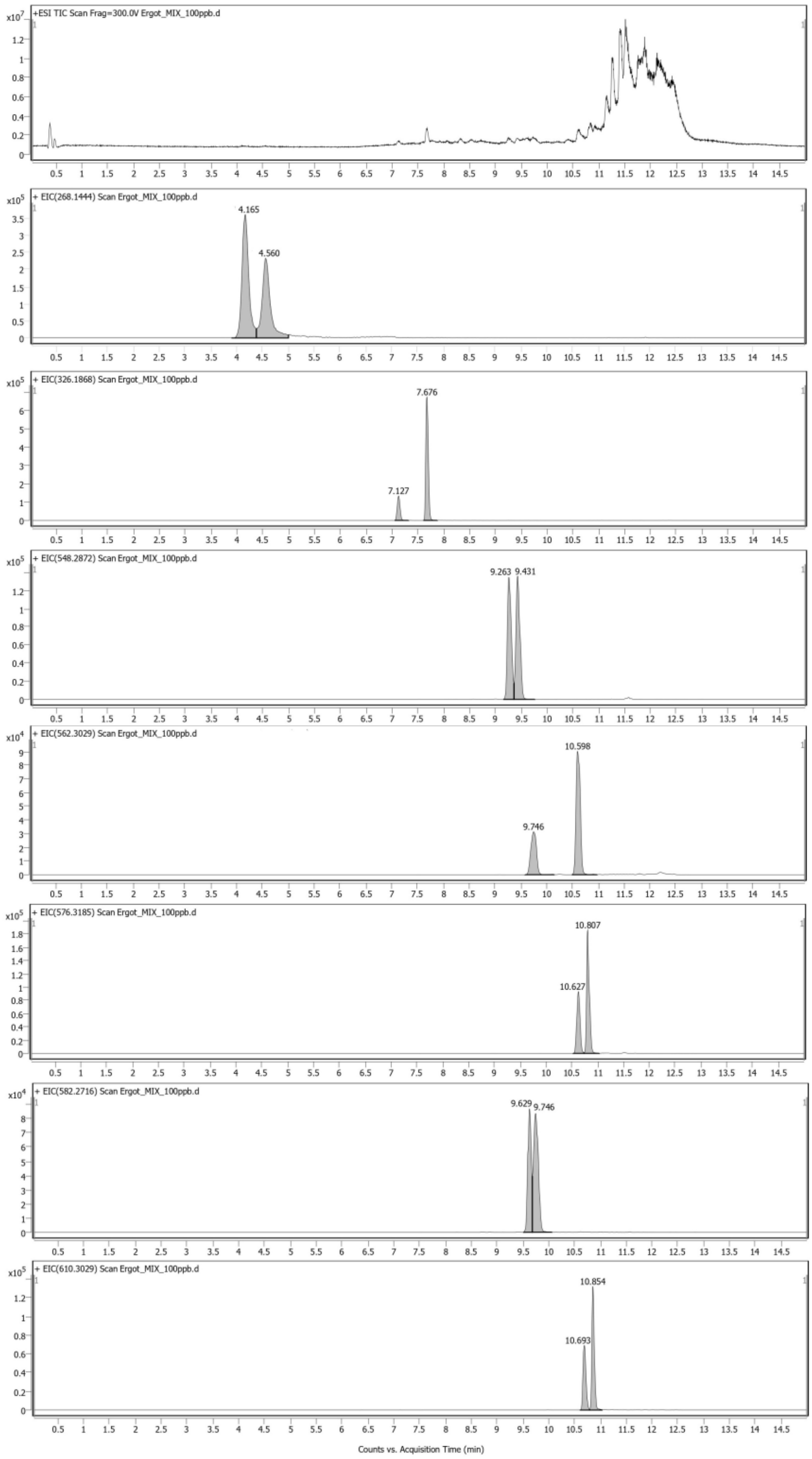

Figure S24. Total ion chromatogram (TIC) and extracted ion chromatograms (EICs) of reference standards.

Table S2. UHPLC/Q-TOF-HRMS quantitation results for LSA and iso-LSA in the extracts and related calculations.

Initial lye pH	Ergot concentration in lye (% w/v)	Reaction time (min)	Dry extract yield of 10 mL aliquot (g)	Mean LSA concentration in extract* (mg g^{-1})	Total LSA in 10 mL aliquot (mg)	Produced LSA (mg g^{-1} of ergot used)	Mean iso-LSA concentration in extract* (mg g^{-1})	Total iso-LSA in 10 mL aliquot (mg)	Produced iso-LSA (mg g^{-1} of ergot used)
7	5	15	0.08	0.38	0.03	0.06	0.42	0.03	0.07
7	5	30	0.10	0.44	0.04	0.09	0.52	0.05	0.11
7	5	60	0.14	0.67	0.09	0.18	0.82	0.11	0.22
7	5	120	0.21	0.27	0.06	0.11	0.39	0.08	0.16
7	10	15	0.19	0.11	0.02	0.02	0.12	0.02	0.02
7	10	30	0.23	0.16	0.04	0.04	0.23	0.05	0.05
7	10	60	0.24	0.26	0.06	0.06	0.33	0.08	0.08
7	10	120	0.29	0.36	0.11	0.11	0.46	0.13	0.13
7	20	15	0.47	0.07	0.03	0.02	0.08	0.04	0.02
7	20	30	0.53	0.08	0.04	0.02	0.13	0.07	0.03
7	20	60	0.55	0.19	0.11	0.05	0.20	0.11	0.05
7	20	120	0.68	0.24	0.16	0.08	0.28	0.19	0.10
10.5	5	15	0.09	0.78	0.07	0.15	0.61	0.06	0.11
10.5	5	30	0.10	1.18	0.12	0.24	0.97	0.10	0.19
10.5	5	60	0.10	1.67	0.18	0.35	1.40	0.15	0.29
10.5	5	120	0.12	1.12	0.14	0.27	0.82	0.10	0.20
10.5	10	15	0.20	0.32	0.07	0.07	0.27	0.05	0.05
10.5	10	30	0.21	0.40	0.08	0.08	0.27	0.06	0.06
10.5	10	60	0.23	0.71	0.16	0.16	0.55	0.13	0.13
10.5	10	120	0.28	0.46	0.13	0.13	0.37	0.10	0.10
10.5	20	15	0.38	0.10	0.04	0.02	0.11	0.04	0.02
10.5	20	30	0.52	0.11	0.06	0.03	0.11	0.06	0.03
10.5	20	60	0.54	0.21	0.11	0.06	0.20	0.11	0.05
10.5	20	120	0.59	0.36	0.21	0.10	0.39	0.23	0.11
11.5	5	15	0.09	0.63	0.06	0.11	0.54	0.05	0.10
11.5	5	30	0.11	0.33	0.04	0.07	0.29	0.03	0.07
11.5	5	60	0.12	0.41	0.05	0.10	0.44	0.05	0.10
11.5	5	120	0.13	0.47	0.06	0.12	0.27	0.04	0.07
11.5	10	15	0.24	0.44	0.11	0.11	0.51	0.13	0.13
11.5	10	30	0.26	0.86	0.22	0.22	0.88	0.22	0.22
11.5	10	60	0.27	1.10	0.29	0.29	1.40	0.38	0.38
11.5	10	120	0.27	1.19	0.32	0.32	1.06	0.29	0.29
11.5	20	15	0.49	0.60	0.29	0.15	0.66	0.32	0.16
11.5	20	30	0.58	0.73	0.42	0.21	0.86	0.49	0.25
11.5	20	60	0.60	0.93	0.56	0.28	1.17	0.70	0.35
11.5	20	120	0.63	1.54	0.96	0.48	1.75	1.10	0.55
12.5	5	15	0.09	4.21	0.38	0.77	4.93	0.45	0.90
12.5	5	30	0.12	3.32	0.38	0.77	2.91	0.34	0.67
12.5	5	60	0.14	2.88	0.39	0.78	3.66	0.50	1.00
12.5	5	120	0.14	1.89	0.27	0.54	1.69	0.24	0.48
12.5	10	15	0.29	1.98	0.57	0.57	2.39	0.68	0.68
12.5	10	30	0.28	2.70	0.76	0.76	3.95	1.12	1.12
12.5	10	60	0.24	3.37	0.81	0.81	3.02	0.72	0.72
12.5	10	120	0.29	2.69	0.77	0.77	2.00	0.57	0.57
12.5	20	15	0.48	2.14	1.02	0.51	2.24	1.07	0.54
12.5	20	30	0.51	1.79	0.91	0.45	2.09	1.06	0.53
12.5	20	60	0.51	2.17	1.11	0.55	2.05	1.05	0.52
12.5	20	120	0.52	2.22	1.15	0.58	2.07	1.08	0.54

*Calculated values represent the means of duplicate experimental runs (n=2). The average relative standard deviation (RSD) across all runs was 10.3% for LSA and 11.3% for iso-LSA, with RSD values as low as 0.45% and 5.24%, respectively, under optimal hydrolysis conditions (5% w/v ergot, lye pH 12.5, 120 min).

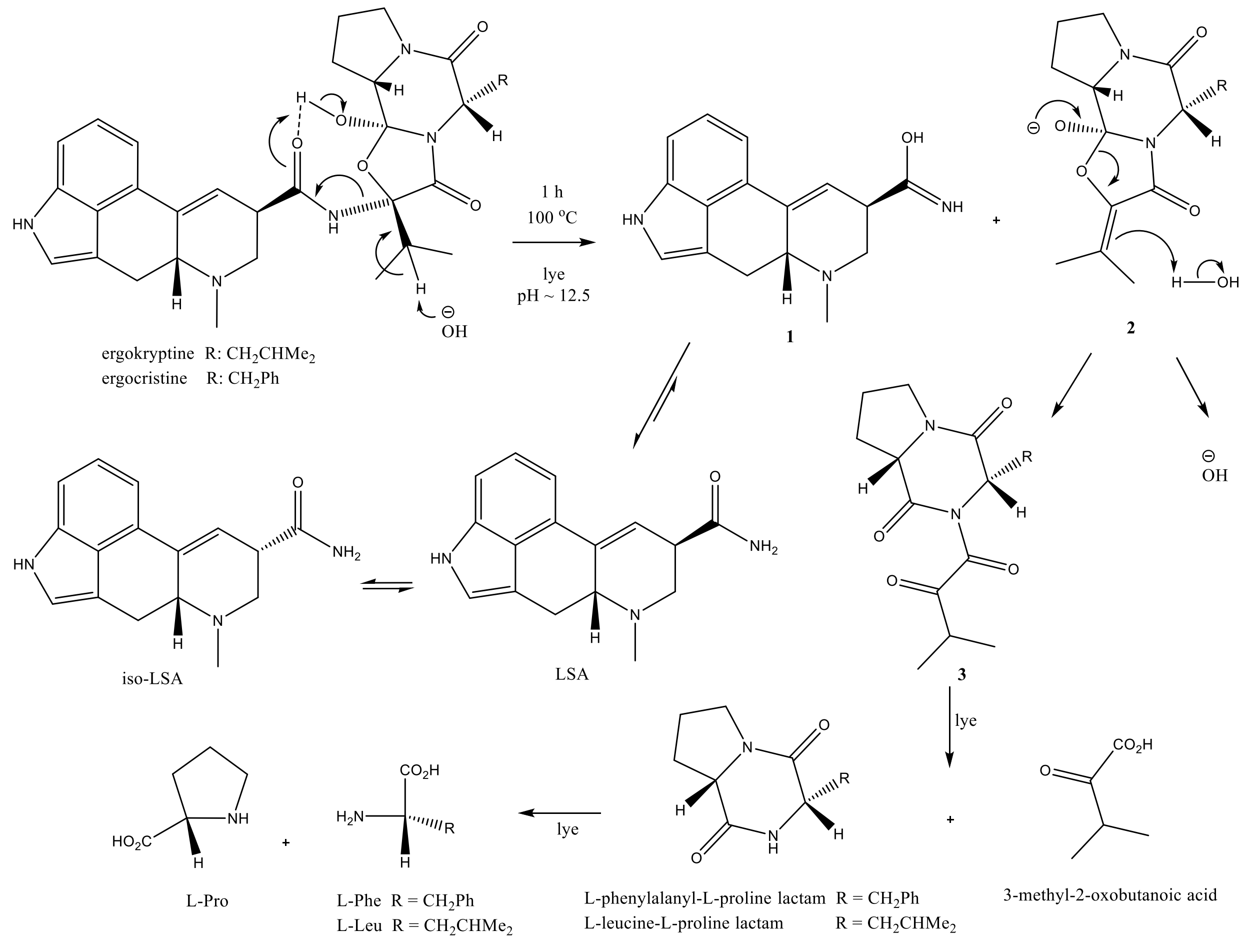

Figure S25. Proposed mechanism of the chemical transformation of the ergopeptines ergokryptine and ergocristine into LSA/iso-LSA and non-toxic secondary by-products. The lye solution hydroxide removes a proton from the isopropyl group, thereby initiating a cascade of reactions, probably more or less simultaneous, involving the intramolecular hydrogen bond and leading to the enol tautomer of LSA/iso-LSA (**1**), which rapidly rearranges to LSA and iso-LSA. The other product of this cleavage (**2**) is a reactive intermediate, which rapidly forms a stable compound (**3**) by extracting a proton from the solvent to regenerate a hydroxide ion. This stable compound should itself be easily hydrolyzed in mild base to produce 3-methyl-2-oxobutanoic acid and lactams, harmless dipeptides, which can further hydrolyze to essential amino acids. The same reactions would apply to C-8 epimers and ergopeptames (adapted and based on literature[5,14,15]).

Table S3. Results of effect tests and parameter estimates, together with their levels of significance, derived from the model equations employed for LSA.

Equation 1				Equation 2				Equation 3			
LSA	Effect Tests	Parameter Estimates			Effect Tests	Parameter Estimates			Effect Tests	Parameter Estimates	
Term	Prob > F	Estimate	Prob>|t|	Source	Prob > F	Estimate	Prob>|t|	Source	Prob > F	Estimate	Prob>|t|
Intercept	-	0.0289	0.5813	Intercept		0.2291	0.0001	Intercept		0.0279	0.5891
X_1	0.0001	0.2703	0.0001	X_1	0.0001	0.2197	0.0001	X_1	0.0001	0.2711	0.0001
X_2	0.2462	-0.0261	0.2462	X_2	0.4546	-0.0192	0.4546	X_2	0.0908	-0.0350	0.0908
X_3	0.0395	0.0495	0.0395	X_3	0.2425	0.0304	0.2425	X_3	0.0431	0.0454	0.0431
X_1^2	0.0001	0.3693	0.0001	X_1*X_2	0.8909	-0.0020	0.8909	X_1^2	0.0001	0.3693	0.0001
X_2^2	0.7599	-0.0125	0.7599	X_1*X_3	0.9902	-0.0438	0.9902	X_2^2	0.7564	-0.0125	0.7564
X_3^2	0.1756	-0.0575	0.1756	X_2*X_3	0.4213	0.0467	0.4213	X_3^2	0.1692	-0.0575	0.1692
X_1*X_2	0.8203	-0.0061	0.8203								
X_1*X_3	0.9838	-0.0006	0.9838								
X_2*X_3	0.1863	0.0354	0.1863								

X1 : lye pH, X2 : ergot powder concentration in the lye, and X3 : reaction time

Table S4. Results of effect tests and parameter estimates, together with their levels of significance, derived from the model equations employed for iso-LSA.

iso-LSA	Effect Tests	Parameter Estimates			Effect Tests	Parameter Estimates			Effect Tests	Parameter Estimates	
Term	Prob > F	Estimate	Prob>|t|	Source	Prob > F	Estimate	Prob>|t|	Source	Prob > F	Estimate	Prob>|t|
Intercept	-	0.0191	0.7700	Intercept		0.2291	0.0001	Intercept		0.0158	0.8096
X_1	0.0001	0.2727	0.0001	X_1	0.0001	0.2197	0.0001	X_1	0.0001	0.2823	0.0001
X_2	0.5359	-0.0173	0.5359	X_2	0.6549	-0.0192	0.6549	X_2	0.2885	-0.0277	0.2885
X_3	0.2229	0.0360	0.2229	X_3	0.5041	0.0304	0.5041	X_3	0.4570	0.0209	0.4570
X_1^2	0.0001	0.4213	0.0001	X_1*X_2	0.9697	-0.0020	0.9697	X_1^2	0.0001	0.4213	0.0001
X_2^2	0.6919	-0.0203	0.6919	X_1*X_3	0.4456	-0.0438	0.4456	X_2^2	0.6934	-0.0203	0.6934
X_3^2	0.1577	-0.0751	0.1577	X_2*X_3	0.3699	0.0467	0.3699	X_3^2	0.1594	-0.0751	0.1594
X_1*X_2	0.9526	-0.0020	0.9526								
X_1*X_3	0.2350	-0.0438	0.2350								
X_2*X_3	0.1636	0.0467	0.1636								

X1 : lye pH, X2 : ergot powder concentration in the lye, and X3 : reaction time

Table S5. Summary of fit based on the model equations applied for LSA and iso-LSA.

	LSA Summary of Fit			iso-LSA Summary of Fit		
	Equation 1	Equation 2	Equation 3	Equation 1	Equation 2	Equation 3
**R^2**	0.830032	0.496444	0.821693	0.781616	0.421537	0.761629
**R^2 Adj**	0.789776	0.422752	0.795599	0.729893	0.336884	0.726745
**Root Mean Square Error**	0.116012	0.192239	0.114394	0.145051	0.227273	0.145894

References

1. Flieger, M. *et al.* Ergochromes: Heretofore neglected side of ergot toxicity. *Toxins* **11**, 439. https://doi.org/10.3390/toxins11080439 (2019).
2. Guerre, P. Ergot alkaloids produced by endophytic fungi of the genus *Epichloë*. *Toxins* **7**, 773–790. https://doi.org/10.3390/toxins7030773 (2015).
3. Hazel, C. M. & Panaccione, D. G. A new species of *Periglandula* symbiotic with the morning glory *Ipomoea tricolor*. *Mycologia* **117**, 602–614. https://doi.org/10.1080/00275514.2025.2483634 (2025).
4. Uhlig, S., Petersen, D., Rolèn, E., Egge-Jacobsen, W. & Vrålstad, T. Ergosedmine, a new peptide ergot alkaloid (ergopeptine) from the ergot fungus, *Claviceps purpurea* parasitizing *Calamagrostis arundinacea*. *Phytochemistry Letters* **4**, 79–85. https://doi.org/10.1016/j.phytol.2010.09.004 (2011).
5. Dewick, P. M. *Medicinal Natural Products: A Biosynthetic Approach*. (J. Wiley, Chichester, 2009).
6. Jakubczyk, D., Cheng, J. Z. & O'Connor, S. E. Biosynthesis of the ergot alkaloids. *Nat. Prod. Rep.* **31**, 1328–1338. https://doi.org/10.1039/C4NP00062E (2014).
7. Arroyo-Manzanares, N., Gámiz-Gracia, L., García-Campaña, A. M., Diana Di Mavungu, J. & De Saeger, S. Ergot alkaloids: Chemistry, biosynthesis, bioactivity, and methods of analysis. in *Fungal Metabolites* (eds Mérillon, J.-M. & Ramawat, K. G.) 887–929. https://doi.org/10.1007/978-3-319-25001-4_1 (Springer International Publishing, Cham, 2017).
8. Flieger, M., Wurst, M. & Shelby, R. Ergot alkaloids — Sources, structures and analytical methods. *Folia Microbiol* **42**, 3–30. https://doi.org/10.1007/BF02898641 (1997).
9. Hofmann, A. Historical view on ergot alkaloids. *Pharmacology* **16**, 1–11. https://doi.org/10.1159/000136803 (1978).
10. Schiff, P. L. Ergot and its alkaloids. *Am J Pharm Educ* **70**, 98. https://doi.org/10.5688/aj700598 (2006).
11. Kidrič, J., Kocjan, D. & Hadži, D. Conformational analysis of the D ring of lysergic acid amides and its bioactive conformation. *Croatica Chemica Acta* **58**, 389–397. (1985).
12. Watkins, C. Let us now praise famous grains. Proceedings of the American Philosophical Society 122, 9–17. https://www.jstor.org/stable/986259 (1978).
13. Rosen, R. M. Hipponax Fr. 48 Dg. and the Eleusinian kykeon. The American Journal of Philology 108, 416. https://doi.org/10.2307/294663 (1987).
14. Wasson, R. G. *et al. The Road to Eleusis: Unveiling the Secret of the Mysteries*. (North Atlantic Books, Berkeley, 2008).
15. Webster, P., Ruck, C. & Perrine, D. Mixing the kykeon. *Eleusis J. Psychoact. Plants Compd* **4**, 3–18. (2000).
